# Ultrafast Preparation of Nonequilibrium FeNi Spinels by Magnetic Induction Heating for Unprecedented Oxygen Evolution Electrocatalysis

**DOI:** 10.34133/2022/9756983

**Published:** 2022-06-01

**Authors:** Bingzhang Lu, Qiming Liu, Chunyang Wang, Zaheer Masood, David J. Morris, Forrest Nichols, Rene Mercado, Peng Zhang, Qingfeng Ge, Huolin L. Xin, Shaowei Chen

**Affiliations:** ^1^Department of Chemistry and Biochemistry, University of California, 1156 High Street, Santa Cruz, California 95064, USA; ^2^Department of Physics and Astronomy, University of California, Irvine, California 92697, USA; ^3^Department of Chemistry and Biochemistry, Southern Illinois University, Carbondale, Illinois 62901, USA; ^4^Department of Chemistry, Dalhousie University, 6274 Coburg Road, Halifax, NS, Canada B3H 4R2

## Abstract

Carbon-supported nanocomposites are attracting particular attention as high-performance, low-cost electrocatalysts for electrochemical water splitting. These are mostly prepared by pyrolysis and hydrothermal procedures that are time-consuming (from hours to days) and typically difficult to produce a nonequilibrium phase. Herein, for the first time ever, we exploit magnetic induction heating-quenching for ultrafast production of carbon-FeNi spinel oxide nanocomposites (within seconds), which exhibit an unprecedentedly high performance towards oxygen evolution reaction (OER), with an ultralow overpotential of only +260 mV to reach the high current density of 100 mA cm^−2^. Experimental and theoretical studies show that the rapid heating and quenching process (ca. 10^3^ K s^−1^) impedes the Ni and Fe phase segregation and produces a Cl-rich surface, both contributing to the remarkable catalytic activity. Results from this study highlight the unique advantage of ultrafast heating/quenching in the structural engineering of functional nanocomposites to achieve high electrocatalytic performance towards important electrochemical reactions.

## 1. Introduction

Design and engineering of low-cost, high-performance catalysts play an important role in the development and advancement of electrochemical energy technologies, such as fuel cells and water electrolyzers [1]. Up to now, a range of functional nanocomposites have been hailed as viable alternatives to the conventional, noble metal-based catalysts, such as doped carbon, and carbon-supported nanoparticles of metals, metal oxides, sulfides, phosphides, and selenides [2–4]. These materials are typically prepared via the “traditional” thermal methods based on pyrolysis and hydrothermal procedures [5–12]. While these methods are rather facile and effective in sample synthesis, they are energy and time-consuming [13, 14], and the slow heating ramp makes it difficult to produce a nonequilibrium phase within the samples, which might be critical in regulating the electronic structure and hence the electrocatalytic activity [15–18]. In a prior study [19], Holewinski et al. demonstrated that whereas Ag and Co metals were immiscible at equilibrium, AgCo alloy nanoparticles could be obtained by removing the sample from the tube furnace while hot and letting it cool down in ambient instead of following a traditional programmed cooling process and exhibited a remarkable electrocatalytic activity towards oxygen reduction reaction (ORR) unseen for either Ag or Co metals alone. Li et al. [20, 21] showed that the laser ablation method could be exploited for the synthesis of Ag and Ru nanoparticles for high-efficiency water splitting, due to the formation of plenty of stacking faults or grain boundaries that were difficult to produce via conventional methods. Yao et al. [22] developed carbothermal shock synthesis to prepare high-entropy alloy nanoparticles within seconds. Chen et al. [23] adopted flash joule heating to synthesize metastable 1 T phase of MoS_2_ with S vacancies and observed a high efficiency towards hydrogen evolution reaction (HER). In another study [24], Zhang et al. developed a laser scribing method where metal oxide/graphene composites were prepared by simply lasing metal precursors loaded onto a graphene scaffold and exhibited a high performance towards oxygen evolution reaction (OER) likely due to the formation of surface defects. Li et al. [20, 25, 26] successfully used the laser ablation method to prepare a series of water splitting electrocatalysts with oxygen vacancies, faults, and unique morphologies.

Despite the progress, the toolbox for such sample synthesis has been limited, and the range of materials that can be produced and the extent of structural engineering remain narrow. Thus, further development of effective protocols for the synthesis of materials with unprecedented structures and properties is of both fundamental and technological significance [27, 28]. In electrochemical water splitting, OER has been recognized as a major bottleneck that limits the overall performance because of complex reaction pathways and sluggish electron-transfer kinetics [17], and FeNi (oxy)hydroxides and spinel oxides have been extensively studied as viable alternatives to the traditional, noble metal-based commercial catalysts [29–34], where manipulation of the occupation of the e_g_ orbitals of the octahedral metals and/or metal-oxygen covalency represents the leading strategies for further enhancement of the OER activity [30]. This is generally achieved by engineering the spinel components, heterometal doping, and introduction of oxygen vacancies [25, 31, 35, 36]. Phase segregation of Fe and Ni in the spinels has been believed to be the leading cause of the apparent loss of the electrocatalytic activity [37]. Yet, such segregation is inevitable for samples prepared via a “tedious” conventional thermal procedure as it is energetically favorable. In addition, residual heteroanions (e.g., Cl) adsorbed on or doped into the surface of Fe, Co, and Ni (hydro/oxyhydro) oxides may play a significant role in OER electrocatalysis [38–40]. Yet the impacts of such anion impurities have remained largely ignored, although most pyrolytically prepared spinel oxides are derived from iron and nickel chlorides. These issues can be addressed with the development of appropriate synthetic methods where nonequilibrium structures can be produced with reduced phase segregation and a remarkable concentration of anion impurities. This is the primary motivation of the present study.

Herein, we report an ultrafast heating/quenching method based on magnetic induction heating/rapid quenching (MIHRQ) [41] to prepare carbon-supported FeNi spinel composites (within seconds), which exhibit a clear mixing of the Ni and Fe phases and a Cl-rich surface, in contrast to the control samples that are prepared by prolonged heating and/or natural cooling to the ambient. In electrochemical measurements, the former displays an outstanding electrocatalytic performance towards OER, with an ultralow overpotential of only +260 mV to reach the high current density of 100 mA cm^−2^, due to the formation of a nonequilibrium structure that is optimal for the adsorption of key reaction intermediates and eventual production of oxygen. The enhanced performance of the catalyst is also confirmed by results of first principle calculations. These results highlight the unique potential of MIHRQ in the deliberate production of nonequilibrium features in composite electrocatalysts. Notably, such an unprecedented tool can be readily extended to the preparation of a wide range of functional nanocomposites for diverse applications [42–47].

## 2. Results and Discussion

### 2.1. Magnetic Induction Heating-Rapid Quenching

**Figure 1:**
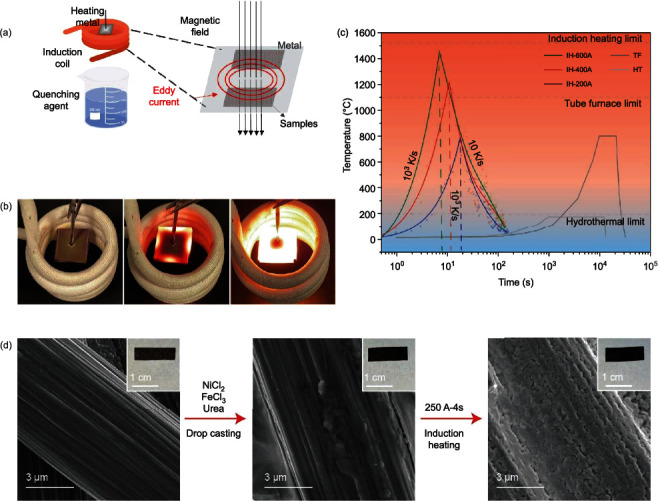
Sample preparation by MIHRQ. (a) Schematic illustration of magnetic induction heating-quenching for material preparation. (b) Photographs of induction heating with a solenoid current at (left) 200 A, (middle) 400 A, and (right) 600 A for 4 s, respectively. (c) Variations of temperature versus time of magnetic induction heating, traditional hydrothermal heating, and pyrolysis. The dash lines indicate the cases with the quenching process. ‘IH,' ‘TF,' and ‘HT' are short for induction heating, tube furnace, and hydrothermal, respectively. (d) SEM images of the NiFeO-250-4 sample at different preparation stages. The insert images are the corresponding photographs of the electrodes. The carbon paper can be seen to become darkened after the deposition of the metal salt precursors, and subsequent induction heating and rapid quenching leads to a pitch-black appearance of the carbon paper, with particulates formed onto the carbon fibers.

The homemade MIHRQ apparatus is shown in Figure 1(a). A four-turn induction solenoid was twisted at a diameter of 5 cm, under which was placed a beaker containing ethanol and dry ice (-78°C) as the quenching agent. Experimentally, a calculated amount of the metal precursors (i.e., FeCl_3_ and NiCl_2_) was dropcast onto a piece of carbon paper (1.5 cm × 0.5 cm), which was then sandwiched between two rectangular iron sheets (2.5 cm × 2.5 cm × 0.01 cm). An iron nail was inserted into the center of the iron sheets and clamped to hold the assembly, which was placed in the center of the induction solenoid (Figure 1(b)). When a high frequency (30 kHz) current was passed to the solenoid, a strong magnetic field was produced, which instantly generated a strong Eddy current in the iron sheets and thus heated the sample rapidly to a high temperature. The induction current and time can be varied to control the heating temperature (Figure 1(c)). For example, a solenoid current of 200 A for a heating time of 4 s would generate a temperature of 200-300°C, which barely changed the color of the iron sheets (Movie S1). Yet, when the solenoid current was increased to 400 and 600 A, the temperature could reach ca. 600 and 1000°C, respectively, inducing a glowing color of the iron sheets from faint red to white (Figure 1(b), Movie S2). Such ultrafast heating (instant heating rate up to 10^3^ K s^−1^) can not only drastically enhance the time efficiency of sample preparation but more importantly also be exploited for the kinetic control of the materials structures, in contrast to typical pyrolysis or hydrothermal synthesis, which is usually completed within the time frame of hours to even days. After a select period of heating (of the order of seconds), the sample would be dropped into the quenching solution below (Movie S1 and S2) or removed from the solenoid and cooled down naturally in the ambient. This offers an additional control of the materials structures, especially for the production of nonequilibrium features.

A series of samples were prepared with the MIHRQ setup at a controlled induction current (X) for a select period of time (Y) and denoted as FeNiO-X-Y (Figure 1(d)). Control samples were prepared in the same manner except for cooling in ambient, and referred to as FeNiO_NC_-X-Y. Notably, MIHRQ can be extended to the preparation of a wide range of electrocatalysts beyond the FeNi spinel oxides (vide infra).

### 2.2. Structural Characterizations

**Figure 2:**
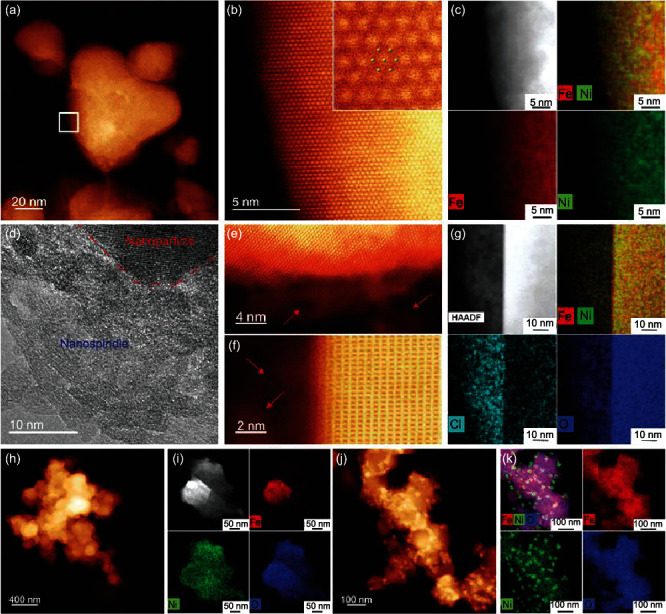
Electron microscopic studies of FeNi spinel oxides. (a) High-angle annular dark-filed scanning transmission electron microscopy (HAADF-STEM) image showing the morphology of FeNiO-250-4. (b) Atomic-resolution HAADF-STEM image and an enlarged image (inset) corresponding to the boxed region in (a) acquired along the <111> zone axis. (c) High-resolution energy-dispersive spectroscopy- (EDS-) based elemental maps of Fe and Ni in a FeNiO-250-4 particle, which features a FeNi spinel structure with no FeNi phase segregation. (d) High-resolution TEM (HRTEM) image of nanospindles in FeNiO-250-4. (e, f) HAADF-STEM images of nanospindles on the edge of FeNiO-250-4 (low-contrast regions), as highlighted by red arrows. (g) EDS mapping images of the interface between nanospindles and nanoparticles in FeNiO_NC_-250-4, which show a Cl-rich surface of the nanospindles. (h) HAADF-STEM images and (i) EDS mapping images of FeNiO_NC_-250-4, where natural cooling leads to the formation of nanoparticles aggregates and obvious FeNi phase segregation. (j) HAADF-STEM and (k) EDS mapping image of FeNiO-250-16, where prolonged heating (higher temperature) leads to the formation of a significant amount of metallic Ni nanoparticles on the FeNi spinel.

High-angle annular dark field-scanning transmission electron microscopy (HAADF-STEM) measurements of the NiFeO-250-4 sample (Figure 1(d)) show the formation of a number of nanoparticles (dia. 20 to 100 nm) in irregular shapes (Figure 2(a)), with the structure consistent with Fe_3-*x*_Ni*_x_*O_4_-type spinel, as observed along the <111> zone axis (Figure 2(b) and Figure S1). The interatomic distance was estimated to be 0.816-0.824 nm (inset to Figure 2(b)), close to that of FeNi spinel oxide (0.835 nm) [48]. Additional STEM images acquired along the <112>, <100>, and<103> zone axes also confirmed the spinel lattice structure (Figure S2–S4). Furthermore, energy-dispersive X-ray spectroscopy- (EDS-) based elemental mapping studies clearly show an even distribution of Fe and Ni within the lattice (Figure 2(c)), suggesting atomic mixing of the Fe and Ni elements and no phase segregation. Notably, spindle-like FeNi oxide nanocrystals can also be found around these particles (Figures 2(d)–2(f) and Figure S5–S7), which feature a chlorine-rich surface (Figure 2(g)), with a Cl concentration of 12% in comparison to under 2% within the particles (Table S1). In fact, the atomic ratio of Fe : Ni : O : Cl in the nanospindles is estimated to be 1.9 : 1 : 4.9 : 1.1, while the overall ratio is close to 4.1 : 1 : 5.8 : 0.22, indicating that the spinel particles were Fe-rich oxide, while the nanospindles likely represent an intermediate phase between the precursors (metal chlorides) and the final spinel crystal (Table S1). Furthermore, it is noticeable that the Fe : Ni ratio is higher than the feeding ratio. This is likely because part of Ni was not fully converted into Ni oxide and washed away during the rapid quenching process.

For FeNiO-250-16 that was prepared via a longer heating time, the Fe : Ni : O : Cl ratio was estimated to be 6.6 : 1 : 8.6 : 0.028 (Table S1), indicative of the formation of a Fe-rich structure that was almost free of Cl. In fact, the nanospindle features, with the unique chlorine rich surface, can only be produced with a short heating time and rapid quenching process.

The control sample, FeNiO_NC_-250-4 that was produced by similar heating but natural cooling in the ambient, exhibited an obviously different morphology (Figure 2(h)), consisting of aggregates of nanoparticles into large chunks. In addition, significant phase segregation occurred within the sample (Figure 2(i)), where the elements of Ni and O appeared to be evenly distributed across the sample, whereas Fe was mostly confined within a small region, suggesting the growth of FeNiO spinel nanocrystals on a nickel oxide scaffold. This is consistent with the sample atomic ratio of Fe : Ni : O : Cl = 0.15 : 1 : 2.3 : 0.036. The fact that the sample was markedly richer in nickel was likely due to higher thermal volatility of the iron compounds [49], where the enhanced loss of Fe was facilitated by the relatively slow cooling (about 10 K s^−1^). Similar phase segregation was also observed with a prolonged heating time (e.g., FeNiO-250-16), where the temperature could reach ca. 1000°C (Figures 2(j) and 2(k) and Figure S8). Note that such nonhomogeneous segregation is actually the equilibrium state at high temperatures based on the phase diagram of Fe-Ni-O_2_ (FToxid database and FactSage) [50], which can be rationally avoided by rapid heating and quenching as manifested with FeNiO-250-4.

**Figure 3:**
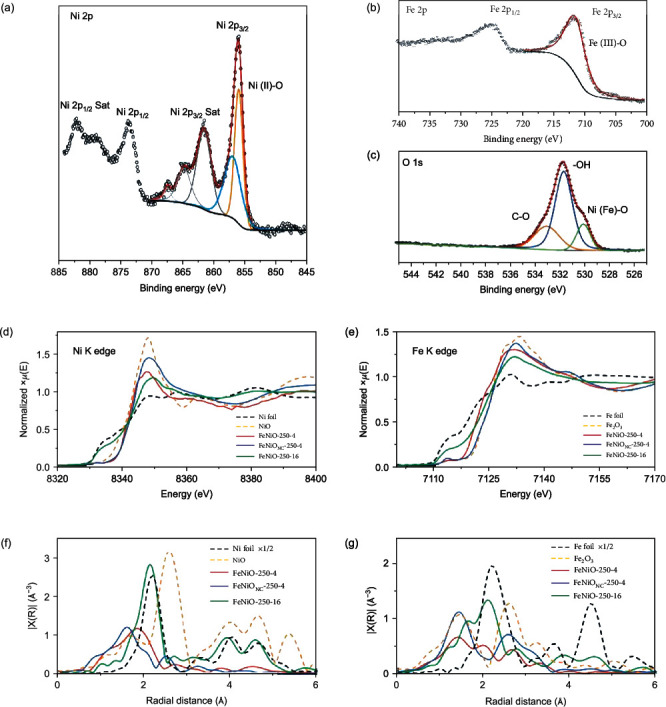
X-ray characterizations of FeNi spinel oxides. High-resolution X-ray photoelectron spectroscopy (XPS) scans of the (a) Ni 2p, (b) Fe 2p, and (c) O 1 s electrons of FeNiO-250-4. (d) Ni K-edge and (e) Fe K-edge X-ray absorption near-edge structure spectra (XANES) of FeNiO-250-4, FeNiO_NC_-250-4, FeNiO-250-16, and reference samples (Ni/Fe foil, NiO, and Fe_2_O_3_), along with the corresponding Fourier transformed extended X-ray absorption fine structure spectra (FT-EXAFS) of (f) Ni and (g) Fe. Note that the Ni-Ni path (2.57 Å) in NiO is essentially absent in FeNiO-250-4 and FeNiO_NC_-250-4, and FeNiO_NC_-250-4 exhibits a first main peak at 1.58 Å, very close to the Ni-O bonds of the NiO reference (1.64 Å), in comparison to 1.83 Å for FeNiO-250-4, possibly because of strong interactions with Cl atoms.

The material structures were further characterized by X-ray photoelectron spectroscopy (XPS) and X-ray absorption spectroscopy (XAS) measurements.  Figure 3(a) depicts the high-resolution XPS scan of the Ni 2p electrons of FeNiO-250-4, where two peaks can be resolved at 855.9 and 856.9 eV for the Ni(II) 3p_3/2_ electrons, suggesting the formation of five and six oxygen-coordinated Ni atoms on the surface (i.e., Ni(OH)_2_), respectively [51], since no NiO species (binding energy around 854.7 eV) was detected [52]. A single peak was resolved at 711.2 eV in the Fe 2p scan (Figure 3(b)), due to the Fe(III) 3p_3/2_ electrons [53], whereas three peaks were deconvoluted in the O 1 s spectrum (Figure 3(d)) at 531.5 eV for hydroxide, 529.9 eV for metal-O, and 533.0 eV for C-O [54]. These observations are consistent with the formation of FeNiO spinel lattices (vide infra). Deconvolution of the Cl 2p spectrum (Figure S9) yields two peaks at 198.3 and 199.9 eV, due to the 2p_3/2_ and 2p_1/2_ electrons of metal-Cl, respectively [55]. Taken together, these results suggest that the FeNiO-250-4 sample surface was mostly terminated with OH and Cl groups, in good agreement with results from the TEM and EDS measurements.

The XPS spectra of the FeNiO_NC_-250-4 and FeNiO-250-16 samples are shown in Figure S10–S12. The Fe 2p spectra showed only a rather insignificant difference among the sample series (Figure S11), likely because of the high thermal activity of FeCl_3_ and the facile formation of Fe oxide. Additionally, FeNiO-250-4 and FeNiO_NC_-250-4 exhibited a very similar Ni 2p profile with the Ni(II) 2p_3/2_ binding energy at 855.9 and 856.9 eV, which was somewhat lower for FeNiO-250-16 (855.6 and 856.75 eV, Figure S10). Furthermore, in the O 1 s spectra (Figure S12), the Ni(Fe)-O peaks (ca. 530 eV) of FeNiO_NC_-250-4 and FeNiO-250-16 are significantly larger than that of FeNiO-250-4, indicating increasing contributions from bulk oxides.

Further oxidation state and structural insights were obtained by XAS measurements.  Figure 3(d) depicts the Ni K-edge X-ray absorption near edge structure (XANES) spectra of the sample series, where the absorption edge intensity can be seen to vary in the order of Ni foil<FeNiO-250-16<FeNiO-250-4<FeNiO_NC_-250-4<NiO, suggesting that the Ni valence state in the three FeNiO samples was in the intermediate between those of metallic Ni and Ni^2+^. A similar trend can be observed in the Fe K-edge XANES in Figure 3(e), where all samples show a clear deviation from that of Fe foil, with the absorption edge intensity varying in the order of Fe foil<FeNiO-250-16<FeNiO-250-4<FeNiO_NC_-250-4<Fe_2_O_3_, confirming that the Fe valence state in the three FeNiO samples was in the intermediate between those of metallic Fe and Fe^3+^. Importantly, the Ni and Fe elements of FeNiO-250-4 can be seen to possess an average oxidation state between those of FeNiO_NC_-250-4 and FeNiO-250-16, likely due to reduced carbothermal effects by the rapid heating and quenching process [22].

Further insights into the bonding configurations of the metal centers were obtained from the extended X-ray absorption fine structure (EXAFS) results. Fitting of the FT-EXAFS data (Figure 3(f), Figure S13, and Table S2–S4) show that FeNiO-250-4 actually possessed Ni-O bonds with a bond length of 2.03 Å, somewhat smaller than those of FeNiO_NC_-250-4 (2.05 Å) and rock salt NiO (2.09 Å) [56]. This is consistent with the phase segregation in FeNiO_NC_-250-4 (vide ante). Meanwhile, the Ni-Cl path in FeNiO-250-4 was found to possess a coordination number (CN) of 3 and an average bond length of 2.40 Å, slightly larger than Ni-O (CN = 2.7). Due to the low cooling rate, a severe Cl loss occurred with FeNiO_NC_-250-4 leading to a low CN of 1.5, while Ni-O showed a CN of 4.7, consistent with the absence of nanospindles in TEM measurements (Figure 2). The profile of FeNiO-250-16 is almost identical to that of Ni foil with a main peak at 2.13 Å for the Ni-Ni path. The Fe EXAFS profile of FeNiO-250-4 (Figure 3(g)) shows three major peaks at 1.38, 1.96, and 2.63 Å, due to Fe-O, Fe-Cl, and second-shell Fe-Fe/Ni bonds, respectively. Yet, the feature of Fe-Cl diminished in both FeNiO_NC_-250-4 and FeNiO-250-16. FeNiO-250-16 displayed a shorter Fe-Fe/Ni bond length (2.57 Å) than FeNiO-250-4 (3.01 Å) and FeNiO_NC_-250-4 (3.04 Å), suggesting a possible transition from spinel structure to metallic Fe [57].

In summary, results from these characterization measurements show that prolonged heating and slow cooling facilitated the O and Cl loss for the spinel samples. Prolonged heating also promoted phase segregation of Ni into rock salt NiO or metallic form. With a deliberate control of the heating time and cooling rate, two key nonequilibrium features of the FeNiO spinel nanoparticles can be achieved, minimal FeNi phase segregation and formation of a Cl-rich surface, both critical in OER electrocatalysis (details below).

### 2.3. Electrocatalytic Activity

**Figure 4:**
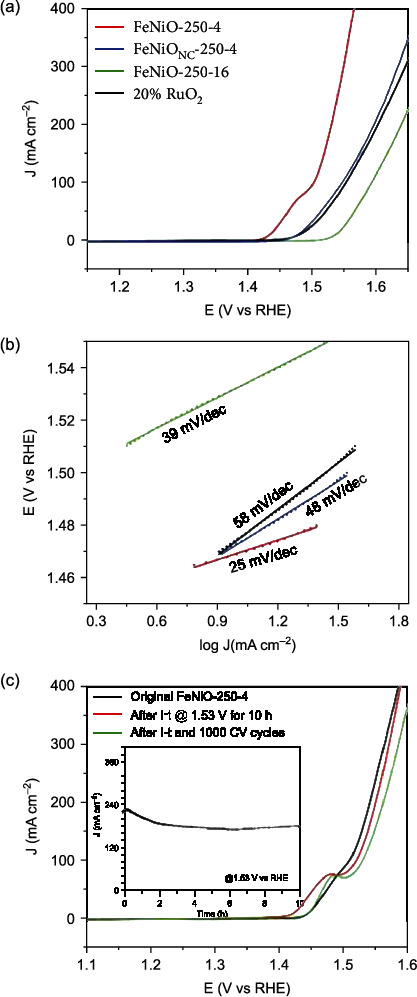
Electrochemical activities. (a) LSV curves and (b) the corresponding Tafel plots of FeNiO-250-4, FeNiO_NC_-250-4, FeNiO-250-16, and 20% RuO_2_/C in 1 M KOH. (c) Polarization curves of FeNiO-250-4 in the first scan, after 10 h's stability tests at +1.53 V (the corresponding i-t curve is shown in the figure inset) and after additional 1000 CV cycles within the potential range of +1.20 to +1.65 V at the scan rate of 10 mV s^−1^.

The FeNiO-250-4 sample that possessed a unique FeNi oxide spinel with Cl-rich surface nanospindles exhibited a remarkably high activity towards OER. From the polarization curves in Figure 4(a), FeNiO-250-4 reached the high current density of 100 mA cm^−2^ in 1 M KOH at an ultralow potential of +1.49 V vs. reversible hydrogen electrode (RHE) (corresponding to an overpotential, *η*_100_, of only +260 mV), in comparison to +1.54 V for FeNiO_NC_-250-4, +1.60 V for FeNiO-250-16, and +1.55 V for commercial 20% RuO_2_. FeNiO-250-4 also displayed a Tafel slope of only 25 mV dec^−1^ markedly lower than the rest of the sample series, 39 mV dec^−1^ for FeNiO-250-16, 48 mV dec^−1^ for FeNiO_NC_-250-4, and 58 mV dec^−1^ for commercial RuO_2_ (Figure 4(b)). In addition, at 100% iR compensation, FeNiO-250-4 can even produce an exceedingly high current density of 1 A cm^−2^ at only +1.64 V (Figure S14), which represents an unprecedentedly high activity among the leading FeNi oxide-based OER electrocatalysts reported in recent studies (Table S5 and Movie S3).

To quantify the electrochemical surface area (ECSA), voltametric measurements were also carried out in the nonfaradaic region of +0.9 to +1.1 V (Figure S15), and FeNiO-250-4 can be seen to exhibit the highest ECSA of 23.06 cm^2^, as compared to 15.75 cm^2^ for FeNiO_NC_-250-4 and 17.06 cm^2^ for FeNiO-250-16 (Table S6). When the OER currents were normalized to the respective ECSA, FeNiO-250-4 remained the best among the series (Figure S16a), reaching a current density of 10 mA cm^−2^ at +1.64 V. The corresponding turnover frequency (TOF) is depicted in Figure S16b at up to 0.21 s^−1^ for FeNiO-250-4, which was at least one order of magnitude higher than those of FeNiO_NC_-250-4 and FeNiO-250-16.

In fact, the FeNiO-250-4 sample represents the optimal condition (Figure S17). It also shows excellent stability. At the applied potential of +1.53 V, over 80% of the initial current was retained even after 10 h's continuous operation (Figure 4(c) inset), which is one of the best performances as compared to the state of the art [37]. The corresponding OER polarization curve showed an anodic shift of only 10 mV. When the electrode was subject to additional 1,000 cyclic voltammetric (CV) scans between +1.20 and +1.65 V, the subsequent polarization curve exhibited a further anodic shift of only 10 mV (Figure 4(c)).

Notably, XPS measurements of the electrocatalyst after the stability test (Figure S18) showed that the Fe : Ni ratio decreased to 1 : 1.8, as compared to that of the as-prepared sample (4.1 : 1, Table S1). This indicates that part of Fe was leached away during the prolonged OER process, which is commonly observed with relevant catalysts [37]. However, Cl remained detectable, and the relative ratio to Fe and Ni (1 : 3) is consistent with that of the pristine sample. Furthermore, in EDS-based elemental mapping analysis (Figure S19), a homogeneous distribution of Fe and Ni, along with Cl, can be readily observed after the stability tests; and the atomic ratio of Fe to Ni was estimated to be 1 : 2, consistent with that from XPS measurements. Taken together, these results suggest that the activity decay is largely due to the Fe loss instead of structural change.

### 2.4. Theoretical Study

**Figure 5:**
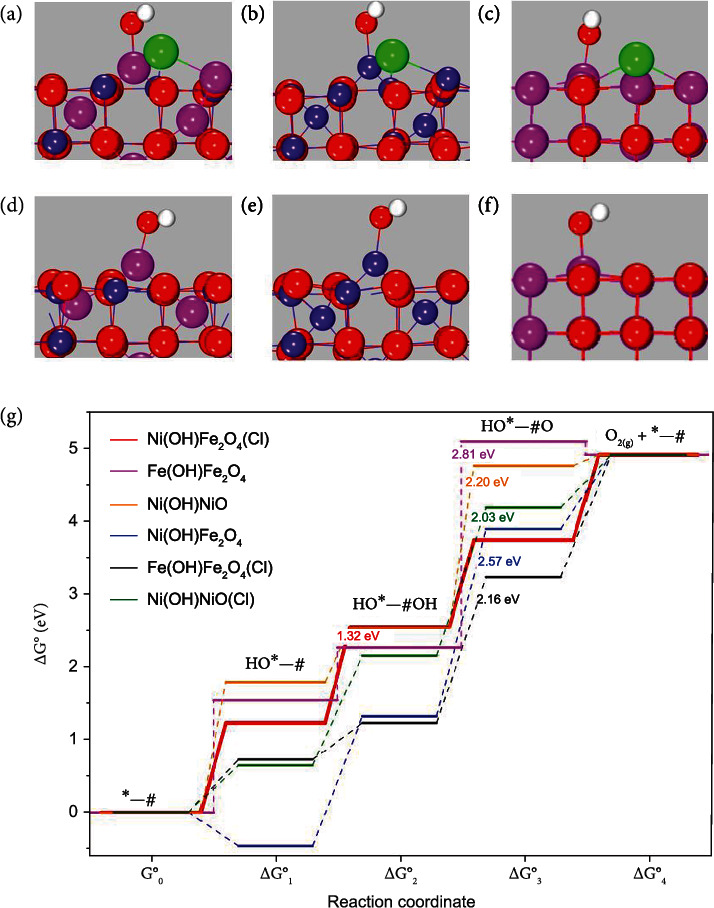
Computational studies of OER energetics. Structural models of (a) Ni(OH)Fe_2_O_4_(Cl), (b) Ni(OH)Fe_2_O_4_, (c) Fe(OH)Fe_2_O_4_(Cl), (d) Fe(OH)Fe_2_O_4_, (e) Ni(OH)NiO(Cl), and (f) Ni(OH)NiO. Free energy profile of OER on Ni(OH)Fe_2_O_4_, Ni(OH)Fe_2_O_4_(Cl), Fe(OH)Fe_2_O_4_, Fe(OH)Fe_2_O_4_(Cl), Ni(OH)NiO and Ni(OH)NiO(Cl). Color codes: green, Cl; red, O; dark blue, Fe; magenta, Ni; white, H.

To unravel the mechanistic origin of the remarkable OER activity observed above with FeNiO-250-4, slab models were built by chlorine substitution of the surface oxygen atom originally located between Fe_oct_ (octahedral site) and Ni_td_ (tetrahedral site) in NiFe_2_O_4_(100) (Figure 5(a)), based on the structural features identified in the above experimental characterization. Free energy calculations indicate that adsorption of OH favors the Ni_td_ sites over the Fe_oct_ sites on the surface, consistent with the XPS results (Figure 3). Consequently, the NiFe_2_O_4_(100) surface with Cl substituting O and Ni_td_ binding an OH (Ni(OH)Fe_2_O_4_(Cl) was used as the model catalyst (Figure 5(a)). Other structures, Ni(OH)Fe_2_O_4_ (Figure 5(b)), Fe(OH)Fe_2_O_4_(Cl) (Figure 5(c)), Fe(OH)Fe_2_O_4_ (Figure 5(d)), Ni(OH)NiO(Cl) (Figure 5(e)), and Ni(OH)NiO (Figure 5(f)), were constructed as comparative references.

A two-site (∗–#) model was adopted to study the OER mechanism (details in the Supporting Information). From the free energy diagram in Figure 5(g), Ni(OH)Fe_2_O_4_(Cl) displays a thermodynamic overpotential of only 90 mV and stands out as the optimal catalyst for OER among all models. The potential-limiting step is the second OH adsorption with a reaction free energy (∆*G*_2_°) of 1.32 eV, while all other steps, including the first OH binding (∆*G*_1_°), O–O coupling (∆*G*_3_°) and O_2_ release (∆*G*_4_°), have a reaction free energy equal to or slightly lower than 1.23 eV. For comparison, Ni(OH)Fe_2_O_4_, with a similar structure but without Cl substitution, shows a very high reaction free energy for the O-O coupling step (∆*G*_3_°), whereas ∆*G*_1_°, ∆*G*_2_°, and ∆*G*_4_° are all markedly below 1.23 eV. The high reaction free energy of O–O coupling accompanied by proton extraction (∆*G*_3_°) indicates that this step is the potential limiting step. These results suggest that incorporation of Cl onto the surface of NiFe_2_O_4_ spinel enhances the OER activity by facilitating O–O bond formation.

To confirm that the active site ensemble on Ni(OH)Fe_2_O_4_(Cl) is unique and responsible for the enhanced activity, we also mapped out the potential energy profiles of OER on the monometal systems of Fe(OH)Fe_2_O_4_, Fe(OH)Fe_2_O_4_(Cl), Ni(OH)NiO, and Ni(OH)NiO(Cl) (Figure 5(g)), where the O–O coupling step, with a respective reaction free energy of 2.81, 2.16, 2.20, and 2.03 eV, remains to be the potential limiting step. These results indicate that these monometal systems, even with Cl substitution, exhibited only a limited OER activity. Therefore, the remarkable activity of FeNiO-250-4 is most likely a synergistic effect of the formation of the metastable FeNi spinel phase and the incorporation of Cl in the surface by substituting surface oxygen atom.

**Figure 6:**
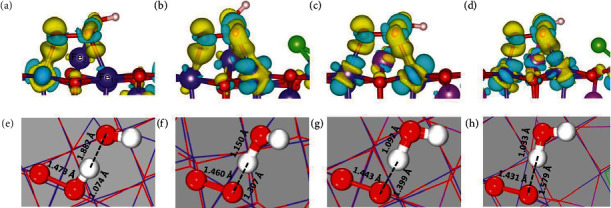
Charge density difference and evolution of bond lengths of two-site models. (a–d) Charge density difference isosurface with a value of 0.01. Light blue corresponds to negative and yellow to positive; (e–h) evolution of bond distances of HO∗—#O species: (a, e) Fe(OH)Fe_2_O_4_, (b, f) Fe(OH)Fe_2_O_4_(Cl), (c, g) Ni(OH)Fe_2_O_4_, and (d, h) Ni(OH)Fe_2_O_4_(Cl). Fe_1_ is next to Cl, and Fe_2_ is away. Color codes: green, Cl; red, O; dark blue, Fe; magenta, Ni; white, H.

To understand the enhanced OER activity in Ni(OH)Fe_2_O_4_(Cl), we tracked the charge density differences and compared the bond distances of the O∗–#OH species on Fe(OH)Fe_2_O_4_, Fe(OH)Fe_2_O_4_(Cl), Ni(OH)Fe_2_O_4_, and Ni(OH)Fe_2_O_4_(Cl). For O∗–#OH adsorbed on Fe(OH)Fe_2_O_4_ (Figure 6(a)), there is no observable charge density redistribution at the Fe_1_ site (adjacent to Cl) but a significant electron depletion is observed on Fe_2_ (away from Cl). With the introduction of Cl (Figure 6(b)), charge redistribution at Fe_1_ is clearly visible whereas the electron density redistribution at Fe_2_ is minimal. The electron density redistribution is believed to stabilize the O–O species on Fe(OH)Fe_2_O_4_(Cl) as a result of losing the proton by O-O--H. Replacing Fe with Ni (Figure 6(c)) results in a relatively uniform charge density redistribution at both Fe_1_ and Fe_2_ sites and further stabilized the O–O species on Ni(OH)Fe_2_O_4_. Therefore, the presence of Cl and Ni (Figure 6(d)) strengthens bonding between the O∗–#OH species and lowers the reaction free energy of the O–O coupling step. Charge redistribution at the Fe_1_ and Fe_2_ sites is also reflected in part in the decrease of Bader charge of the O–O pair, which was -1.2|e|, -1.14|e|, -1.04|e|, and -1.0|e| for the O–O pair adsorbed on Fe(OH)Fe_2_O_4_, Fe(OH)Fe_2_O_4_(Cl), Ni(OH)Fe_2_O_4_, and Ni(OH)Fe_2_O_4_(Cl), respectively. A decreased negative charge value indicates an increase of acidity of O∗–#OH, which benefits the proton transfer from O∗–#OH to the OH adsorbed on Ni_td_ or Fe_td_ (Figures 6(e)–6(h)). A complete proton transfer from HO∗—#O species to the OH facilitates the O–O bond formation, and the O-H distance in HO∗—#O increases from 1.074 Å on Fe(OH)Fe_2_O_4_ to 1.307 Å on Fe(OH)Fe_2_O_4_(Cl), further to 1.399 Å on Ni(OH)Fe_2_O_4_, and finally to 1.579 Å on Ni(OH)Fe_2_O_4_(Cl) (Figures 6(e)–6(h)). Correspondingly, this increasingly detached proton approaches the OH on Ni_td_ or Fe_td_ to form H_2_O. The loss of H from HO∗—#O also strengthens the O–O bond, as the bond distance decreases from 1.473 Å on Fe(OH)Fe_2_O_4_ to 1.431 Å on Ni(OH)Fe_2_O_4_(Cl). In summary, the presence of Ni and Cl in the catalyst synergistically stabilizes the O–O species while facilitates proton transfer from HO∗—#O to the adjacent OH, resulting in a much reduced free energy barrier for the O–O coupling step.

From the Fe_1_ density of state (DOS) plots (Figure S20), it is evident that the presence of Cl in Fe(OH)Fe_2_O_4_(Cl) and Ni(OH)Fe_2_O_4_(Cl) shifts the occupied d states closer to the Fermi level. For Ni(OH)Fe_2_O_4_(Cl), these states remain occupied up to the O–O coupling step (Figure S20m–S20p). The upward shift of the occupied d states results in an increased reactivity of the Cl and Ni containing catalysts.

## 3. Conclusions

In summary, the MIHRQ method was successfully developed and exploited for the ultrafast fabrication of metal oxide spinel nanostructures. Using NiCl_2_ and FeCl_3_ as the precursors, FeNi oxide spinels were obtained by heating at controlled currents within seconds and exhibited an even mixing of the Ni and Fe elements and a Cl-rich surface, in sharp contrast to samples prepared at prolonged heating and/or natural cooling in the ambient. The best sample FeNiO-250-4 needed an overpotential of only +260 mV to reach the high current density of 100 mA cm^−2^ and exhibited significant stability in alkaline media. Such a remarkable activity was attributed to the unique metastable structure that facilitated the adsorption of key reaction intermediates and O-O coupling, a major limiting step in OER. Results from this study highlight the unique advantages of MIHRQ in the rapid production of unprecedented material structures that are unattainable in conventional thermal processes for enhanced electrocatalytic performance and potential applications in the structural engineering of a diverse range of materials (Figure S21).

## 4. Materials and Methods

### 4.1. Sample Preparation

Carbon paper (TGP-H-060 from Toray Industries, Inc.) was cut into 1 cm × 1.5 cm pieces. A solution was prepared by dissolving 40 mg of NiCl_2_•6H_2_O, 10 mg of FeCl_3_, and 0.8 g of urea into 10 mL of water (supplied with a Barnstead Nanopure Water System, 18.2 M*Ω* cm). 100 mL of the solution was dropcast onto the carbon paper, which was then dried at ambient temperature and sandwiched between two iron sheets of 2.5 cm × 2.5 cm × 0.01 cm. The assembly was then placed in the center of a four-turn induction coil with a diameter of 5 cm, and magnetic induction heating was carried out at a controlled current (*X* = 100–600 A) for a select heating time (*Y* = 2–16 s), when the sample was dropped into an ethanol-dry ice solution (-78°C) placed underneath the induction coil for rapid quenching (caution: the ethanol must be fully cooled down by dry ice, or it will catch fire). The temperature was measured with an industrial infrared laser thermometer (BTMETER, BT-1500). The corresponding sample was denoted as FeNiO-X-Y.

Control samples were also prepared by removing the sample assembly from the heater and being cooled down in ambient to room temperature. These samples were referred to as FeNiO_NC_-X-Y.

### 4.2. Structural Characterizations

(Scanning) transmission electron microscopy (S)TEM experiments ware conducted with a transmission electron microscope equipped with an X-FEG field-emission source, operated at 200 keV. To perform the high-angle annular dark-field scanning transmission electron microscopy (HAADF-STEM) imaging and energy-dispersive X-ray spectroscopy (EDS) analysis, the samples were first sonicated, dispersed in ethanol, and then deposited onto copper grids for TEM characterization. Scanning electron microscopy (SEM) studies were carried out on FEI Quanta 3D FEG dual beam instrument. X-ray photoelectron spectroscopy (XPS) measurements were performed with a Phi 5400/XPS instrument equipped with an Al K_*α*_ source operated at 350 W and 10^−9^ Torr. X-ray diffraction (XRD) patterns were acquired with a Bruker D8 Advance diffractometer with Cu K_*α*_ radiation (*λ* = 0.15418 nm).

### 4.3. X-Ray Absorption Spectroscopy (XAS) Studies

Fe and Ni K-edge XAS data was collected from the CLS@APS Sector 20-BM beamline at the Advanced Photon Source (operating at 7.0 GeV) in Argonne National Labs, Chicago, IL, USA. Samples were enclosed within a Kapton tape and measured in fluorescence mode simultaneously with each element foil reference. All measurements were conducted at room temperature and ambient pressure. EXAFS data was transformed and normalized into k- and R-space using the Athena program following conventional procedures. A k weighting of 2 was used to obtain all FT-EXAFS spectra. The k-range used for each sample is as follows for Fe: 3.1–9.2 Å^−1^ for FeNiO-250-4, 2.1–9.1 Å^−1^ for FeNiO_NC_-250-4, and 3.3–12.7 Å^−1^ for FeNiO-250-16. For Ni, the k-range used was as follows: 3.0–8.9 Å^−1^ for FeNiO-250-4, 2.9–12.2 Å^−1^ for FeNiO_NC_-250-4, and 2.6–14.4 Å^−1^ for FeNiO-250-16. The R-range used for Fe is as follows: 1.0-3.7 Å for FeNiO-250-4, 1.0–3.6 Å for FeNiO_NC_-250-4, and 1.0–3.4 Å for FeNiO-250-16. The R-range used for Ni is as follows: 1.0-3.5 Å for FeNiO-250-4, 1.0–3.0 Å for FeNiO_NC_-250-4, and 1.0–3.0 Å for FeNiO-250-16. Self-consistent multiple-scattering calculations were performed using the FEFF6 program to obtain the scattering amplitudes and phase-shift functions used to fit various scattering paths with the Artemis program. In the fitting of each sample, the *E*_0_ values were correlated together to minimize the number of independent values, allowing reliable fitting results to be obtained. The *σ*^2^ values were also correlated for some samples.

### 4.4. Electrochemistry

Electrochemical measurements were carried out with a CHI 700e electrochemical workstation in a three-electrode configuration. The prepared carbon paper was fixed onto a graphite electrode holder, with an exposed surface area of 1 cm^2^. A platinum wire was adopted as the counter electrode and a Ag/AgCl in saturated KCl as the reference electrode. The reference electrode was calibrated against a reversible hydrogen electrode (RHE), and all potentials in the present study were referenced to this RHE.

### 4.5. DFT computation

Spin-polarized density functional theory (DFT) calculations were carried out using the VASP (Vienna Ab-Initio Simulation Package) code [58]. Projector-augmented wave (PAW) method [59] with the Perdew-Burke-Ernzerhof (PBE) exchange-correlation functional [60] was used in all calculations. On-site Coulomb interactions were corrected within the DFT+U framework based on Dudarev's approximation [61]. U_eff_ = 4.20 [62] and 6.40 [63] for Fe and Ni, respectively, were used [64]. Plane-wave basis set with a 400 eV energy cutoff provides a balance of accuracy and computational cost. Either quasi-Newton scheme or conjugate gradient algorithm implemented in VASP was used to relax structure until forces are converged to less than -0.03 eV Å^−1^ on unconstrained atoms and self-consistent convergence until 10^−5^ eV. The Gaussian smearing with a *σ* value of 0.05 was used to minimize entropy contribution to free energy. The bulk structure of NiFe_2_O_4_ was taken from JCPDS (JCPDS Card No. 10-0325) and optimized in (4 × 4 × 1) k-point grid sampling of the surface Brillouin zone. The optimized lattice constant of 8.37 Å was found in close agreement with the experimental value of 8.35 Å.

A supercell consisting of five layers of NiFe_2_O_4_ with an exposed (100) surface was constructed from the optimized bulk structure. A vacuum space of 14 Å in *z*-direction was inserted between the slabs, and the atoms in the top three layers were allowed to relax while those in the bottom two layers were fixed at the corresponding bulk position during structural optimization.

Additional details are included in the Supporting Information.

## Data Availability

The data used to support the findings of this study are included within the article.

## Supplementary Materials

Supplementary Materials Additional computational details. Figure S1: crystal structure of FeNi oxide spinel, where blue, pink, and red indicate Fe, Ni, and O atoms, respectively. Figure S2: HAADF-STEM images of FeNi oxide spinel in FeNiO-250-4 along the [112] zone axis. Figure S3: HAADF-STEM images of FeNi oxide spinel in FeNiO-250-4 along the [100] zone axis. Figure S4: HAADF-STEM images of FeNi oxide spinel in FeNiO-250-4 along the [103] zone axis. Figure S5: TEM and electron diffraction studies. Figure S6: EDS-based elemental mapping analysis. Figure S7: EDS-based elemental mapping analysis. Figure S8: XRD patterns of the sample series. Figure S9: XPS spectrum of the Cl 2p electrons of FeNiO-250-4. Figure S10: high-resolution XPS spectra of the Ni 2p electrons of (a) FeNiO_NC_-250-4 and (b) FeNiO-250-16. Figure S11: high-resolution XPS spectra of the Fe 2p electrons of (a) FeNiO_NC_-250-4 and (b) FeNiO-250-16. Figure S12: high-resolution XPS scans of the O 1 s electrons of (a) FeNiO_NC_-250-4 and (b) FeNiO-250-16. Figure S13: EXAFS fitting results. Figure S14: LSV curves of FeNiO-250-4 in 1 M KOH at different levels of iR compensation. Figure S15: CV curves at difference scan rates for (a) FeNiO-250-4, (b) FeNiO_NC_-250-4, and (c) FeNiO-250-16. (d) The variation of the double-layer charging current with potential scan rate. Figure S16: (a) LSV curves normalized to ECSA and (b) TOF curves of FeNiO-250-4, FeNiO_NC_-250-4, and FeNiO-250-16. Figure S17: optimization of OER performance of FeNiO samples. Figure S18: high-resolution XPS spectra of the (a) Ni 2p, (b) Fe 2p, (c) Cl 2p, (d) C 1 s, and (e) O 1 s electrons of FeNiO-250-4 after stability test. Figure S19: SEM image and the corresponding EDS-based elemental mapping analysis of FeNiO-250-4 after stability test. Figure S20: PDOS of the Fe1 atom. Figure S21: additional materials prepared by MIHRQ. Table S1: elemental contents of the sample series based on EDS measurements. Table S2: fitting results of the EXAFS data of FeNiO-250-4. Table S3: fitting results of the EXAFS data of FeNiO_NC_-250-4. Table S4: fitting results of the EXAFS data of FeNiO-250-16. Table S5: comparison with relevant FeNi oxide-based OER catalysts reported in the literature. Table S6: electrochemical surface areas of the sample series. Movie S1: magnetic induction heating at a solenoid current of 200 A for a heating time of 4 s. Movie S2: magnetic induction heating at a solenoid current of 600 A for a heating time of 4 s. Movie S3: oxygen evolution catalyzed by FeNiO-250-4.
