# Supplementary material for: Ultrafast Preparation of Nonequilibrium FeNi Spinels by Magnetic Induction Heating for Unprecedented Oxygen Evolution Electrocatalysis
Source: Research (Wash D C). 2022 Jun 1;2022:9756983. doi: 10.34133/2022/9756983 (PMC9185434; doi:10.34133/2022/9756983)
Supplement: Supplementary Materials — Additional computational details. Figure S1: crystal structure of FeNi oxide spinel, where blue, pink, and red indicate Fe, Ni, and O atoms, respectively. Figure S2: HAADF-STEM images of FeNi oxide spinel in FeNiO-250-4 along the [112] zone axis. Figure S3: HAADF-STEM images of FeNi oxide spinel in FeNiO-250-4 along the [100] zone axis. Figure S4: HAADF-STEM images of FeNi oxide spinel in FeNiO-250-4 along the [103] zone axis. Figure S5: TEM and electron diffraction studies. Figure S6: EDS-based elemental mapping analysis. Figure S7: EDS-based elemental mapping analysis. Figure S8: XRD patterns of the sample series. Figure S9: XPS spectrum of the Cl 2p electrons of FeNiO-250-4. Figure S10: high-resolution XPS spectra of the Ni 2p electrons of (a) FeNiONC-250-4 and (b) FeNiO-250-16. Figure S11: high-resolution XPS spectra of the Fe 2p electrons of (a) FeNiONC-250-4 and (b) FeNiO-250-16. Figure S12: high-resolution XPS scans of the O 1 s electrons of (a) FeNiONC-250-4 and (b) FeNiO-250-16. Figure S13: EXAFS fitting results. Figure S14: LSV curves of FeNiO-250-4 in 1 M KOH at different levels of iR compensation. Figure S15: CV curves at difference scan rates for (a) FeNiO-250-4, (b) FeNiONC-250-4, and (c) FeNiO-250-16. (d) The variation of the double-layer charging current with potential scan rate. Figure S16: (a) LSV curves normalized to ECSA and (b) TOF curves of FeNiO-250-4, FeNiONC-250-4, and FeNiO-250-16. Figure S17: optimization of OER performance of FeNiO samples. Figure S18: high-resolution XPS spectra of the (a) Ni 2p, (b) Fe 2p, (c) Cl 2p, (d) C 1 s, and (e) O 1 s electrons of FeNiO-250-4 after stability test. Figure S19: SEM image and the corresponding EDS-based elemental mapping analysis of FeNiO-250-4 after stability test. Figure S20: PDOS of the Fe1 atom. Figure S21: additional materials prepared by MIHRQ. Table S1: elemental contents of the sample series based on EDS measurements. Table S2: fitting results of the EXAFS data of FeNiO-250-4. Table S3: fitt [file 9756983.f1.zip › SI-Research rev.pdf]

Supporting Information

Ultrafast Preparation of Non-Equilibrium Fe-Ni Spinels by Magnetic Induction Heating for Unprecedented Oxygen Evolution Electrocatalysis

Bingzhang Lu,[1,5,6] Qiming Liu,[1,5] Chunyang Wang,[2] Zaheer Masood,[3] David J. Morris,[4] Forrest Nichols,[1] Rene Mercado,[1] Peng Zhang,[4] Qingfeng Ge,[3,*] Huolin L. Xin,[2,*] and Shaowei Chen[1,*]

[1] Department of Chemistry and Biochemistry, University of California, 1156 High Street, Santa Cruz, California 95064, United States

[2] Department of Physics and Astronomy, University of California, Irvine, California 92697, United States

[3] Department of Chemistry and Biochemistry, Southern Illinois University, Carbondale, Illinois 62901, United States

[4] Department of Chemistry, Dalhousie University, 6274 Coburg Road, Halifax, NS, B3H 4R2, Canada

[5] These authors contributed equally to the work.

[6] Present address: Department of Chemical Engineering, Northwestern University, 633 Clark St, Evanston, Illinois 60208, United States

* E-mail: shaowei@ucsc.edu, qge@chem.siu.edu, huolinx@uci.edu

List of Contents

- Additional computational details
- 21 figures
- 6 tables
- 3 movies

ADDITIONAL COMPUTATIONAL DETAILS

Generally, a single active site mechanism, shown in eq. S1-S4, has been widely used in analyzing oxygen evolution reaction (OER) catalyzed by an oxide catalyst, such as Ni, Co, Fe spinels.

$$* + OH \rightarrow *\text{–}OH \quad (S1)$$

$$*\text{–}OH + OH \rightarrow *\text{–}O + H_2O \quad (S2)$$

$$*\text{–}O + OH \rightarrow *\text{–}OOH \quad (S3)$$

$$*\text{–}OOH + OH \rightarrow O_2 + * + H_2O \quad (S4)$$

On the $NiFe_2O_4$ catalyst, Ni was considered as the active site for OER and the potential limiting step was *-O → *-OOH (eq. S3). This step has a reaction free energy of 2.0 eV, corresponding to a thermodynamic overpotential of ca. 770 mV.[*1*] Another study reported that *-OH → *-O (eq. S2) was the potential limiting step and had a thermodynamic overpotential of 970 mV.[*2*] Obviously, the overpotentials from both studies are significantly higher than the present experimental result, i.e., ca. 200 mV at 1 mA cm^{-2} from Fig. 4a. Based on the scaling relationship between the binding energies of *OH and *OOH, an overpotential less than 0.4 V cannot be achieved by following the single metal site mechanism.[*3*] Therefore, the mechanism based on a single metal site was not believed to contribute to the high activity observed in the present study.

We adopted a mechanism involving two adjacent metal sites, i.e., *—#. According to this mechanism, shown in eq. S5–S8, OER starts by OH binding on the first metal site forming HO*—# (ΔG_1°, eq. S5). This is followed by a second OH binding at the neighboring metal site forming HO*—#OH (ΔG_2°, eq. S6). A stepwise reaction of HO*—#OH with OH (ΔG_3°, eq. S7 and ΔG_4°, eq. S8) releases O_2 and H_2O and completes the cycle.

$$*\text{—}\# + 4(OH^- + h^+) \rightarrow HO*\text{—}\# + 3(OH^- + h^+)^- \qquad \Delta G_1^\circ \quad (S5)$$

$$HO*\text{—}\# + 3(OH^- + h^+) \rightarrow HO*\text{—}\#OH + 2(OH^- + h^+) \qquad \Delta G_2^\circ \quad (S6)$$

$$HO*\text{—}\#OH + 2(OH^- + h^+) \rightarrow HO*\text{—}\#O + H_2O + (OH^- + h^+) \qquad \Delta G_3^\circ \quad (S7)$$

$$HO*\text{—}\#O + H_2O + (OH^- + h^+) \rightarrow *\text{—}\# + O_2 + 2H_2O \qquad \Delta G_4^\circ \quad (S8)$$

The reaction free energies of these steps were calculated according to eq. S5-S8. Based on the calculated reaction free energies, we constructed the reaction free energy profile shown in Figure 5g and determined the thermodynamic overpotential.

The reaction free energy expressions for the reactions described in eq. S5-S8 are:

$$\Delta G_1^\circ = \mu(HO*\text{—}\#) + 3\mu(OH^- + h^+) - \mu(*\text{—}\#) - 4\mu(OH^- + h^+) \quad (S9)$$

$$\Delta G_2^\circ = \mu(HO*\text{—}\#OH) + 2\mu(OH^- + h^+) - \mu(HO*\text{—}\#) - 3\mu(OH^- + h^+) \quad (S10)$$

$$\Delta G_3^\circ = \mu(HO*\text{—}\#O) + \mu(H_2O) + \mu(OH^- + h^+) - \mu(HO*\text{—}\#OH) - 2\mu(OH^- + h^+) \quad (S11)$$

$$\Delta G_4^\circ = 4.92 - (\Delta G_1^\circ + \Delta G_2^\circ + \Delta G_3^\circ) \quad (S12)$$

Since PBE significantly overestimates $\mu(O_{2(g)})$, ΔG_4° in eq. S12 was computed on the basis of the experimental reaction free energy of 4.92 eV for $2H_2O_{(l)} \rightarrow 2H_{2(g)} + O_{2(g)}$. The chemical potential of OH, i.e., $\mu(OH^- + h^+)$ was computed using the approach developed by Tang and Ge[*4*] based on CHE[*5*]. Free energies of all intermediates were determined using $G^\circ = E_{elect}^\circ - TS + ZPE$. E_{elect}° was obtained from DFT calculations, whereas the contributions of TS and ZPE were computed from frequency calculations in which adsorbate together with the atoms in the topmost layer were allowed to move.

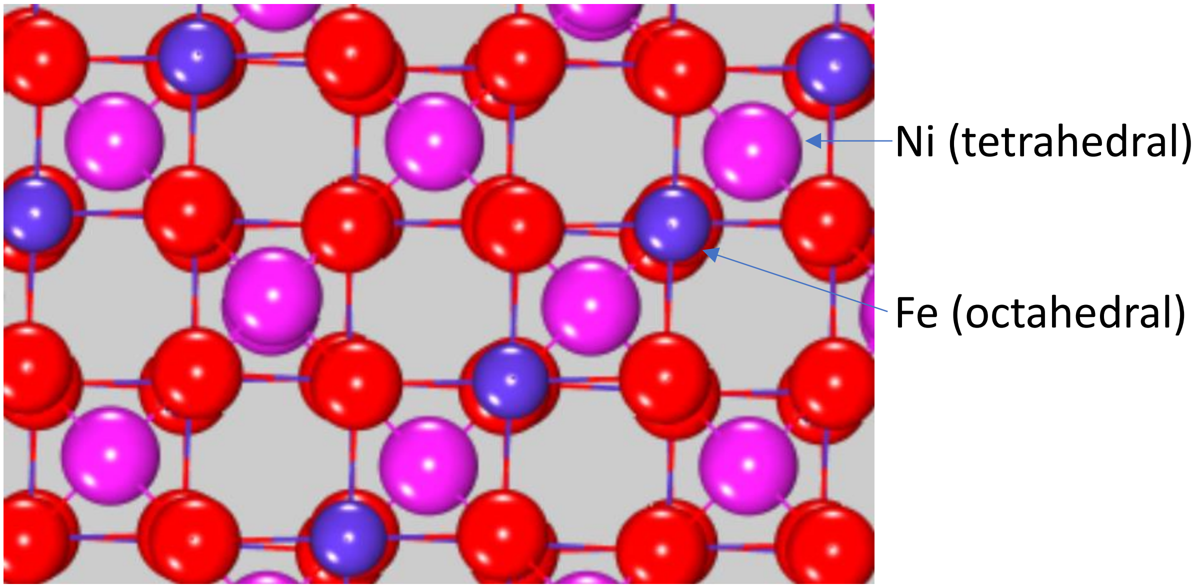

Figure S1. Crystal structure of Fe-Ni oxide spinel, where blue, pink and red indicate Fe, Ni and O atoms, respectively.

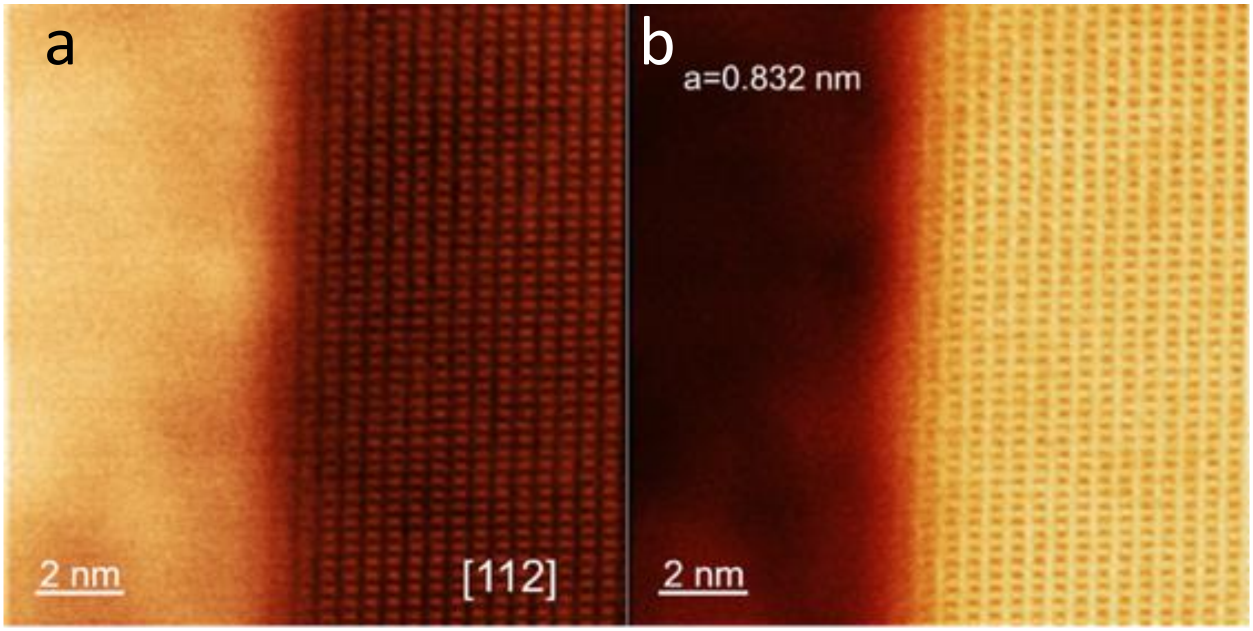

Figure S2. HAADF-STEM images of Fe-Ni oxide spinel in FeNiO-250-4 along the [112] zone axis

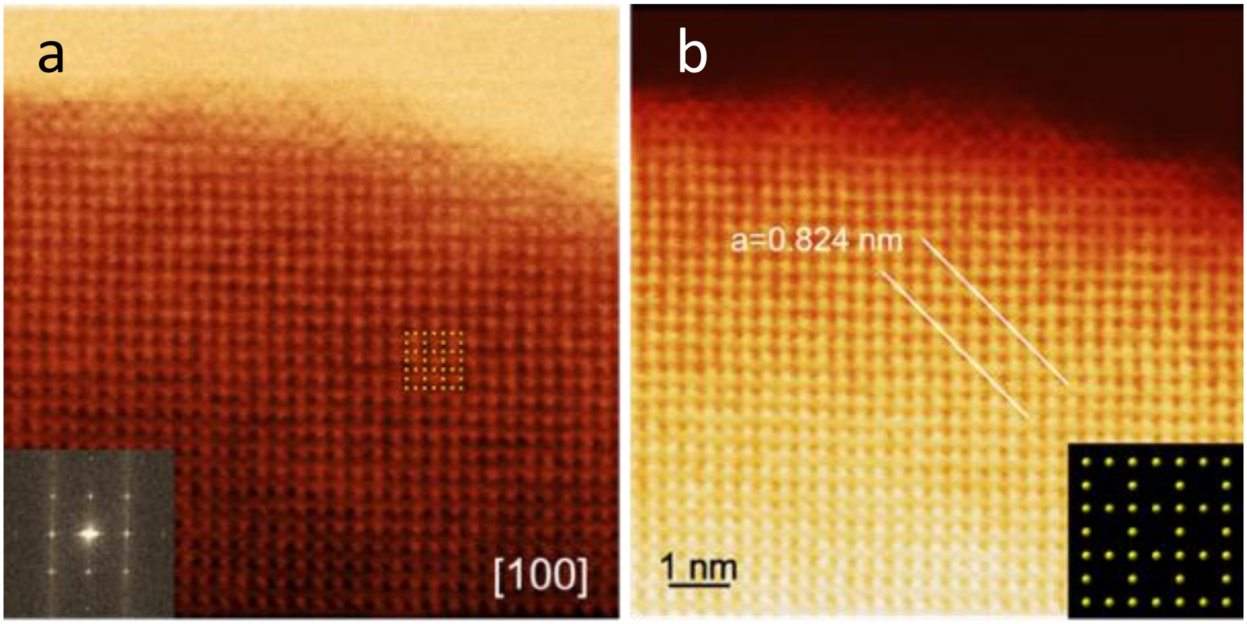

Figure S3. HAADF-STEM images of Fe-Ni oxide spinel in FeNiO-250-4 along the [100] zone axis. Inset to panel (a) is the FFT patterns, whereas inset to panel (b) is a structural model of the atomic arrangement.

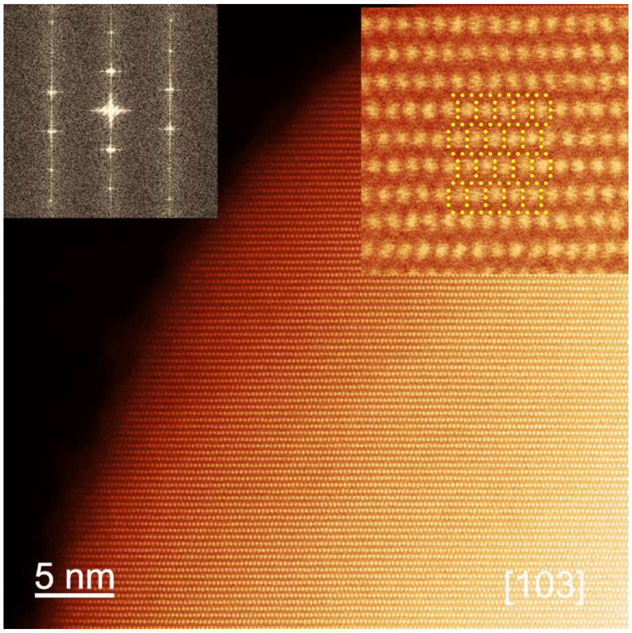

Figure S4. HAADF-STEM images of Fe-Ni oxide spinel in FeNiO-250-4 along the [103] zone axis. Left inset is the FFT patterns, and the right inset is a magnified image highlighting the atomic arrangement.

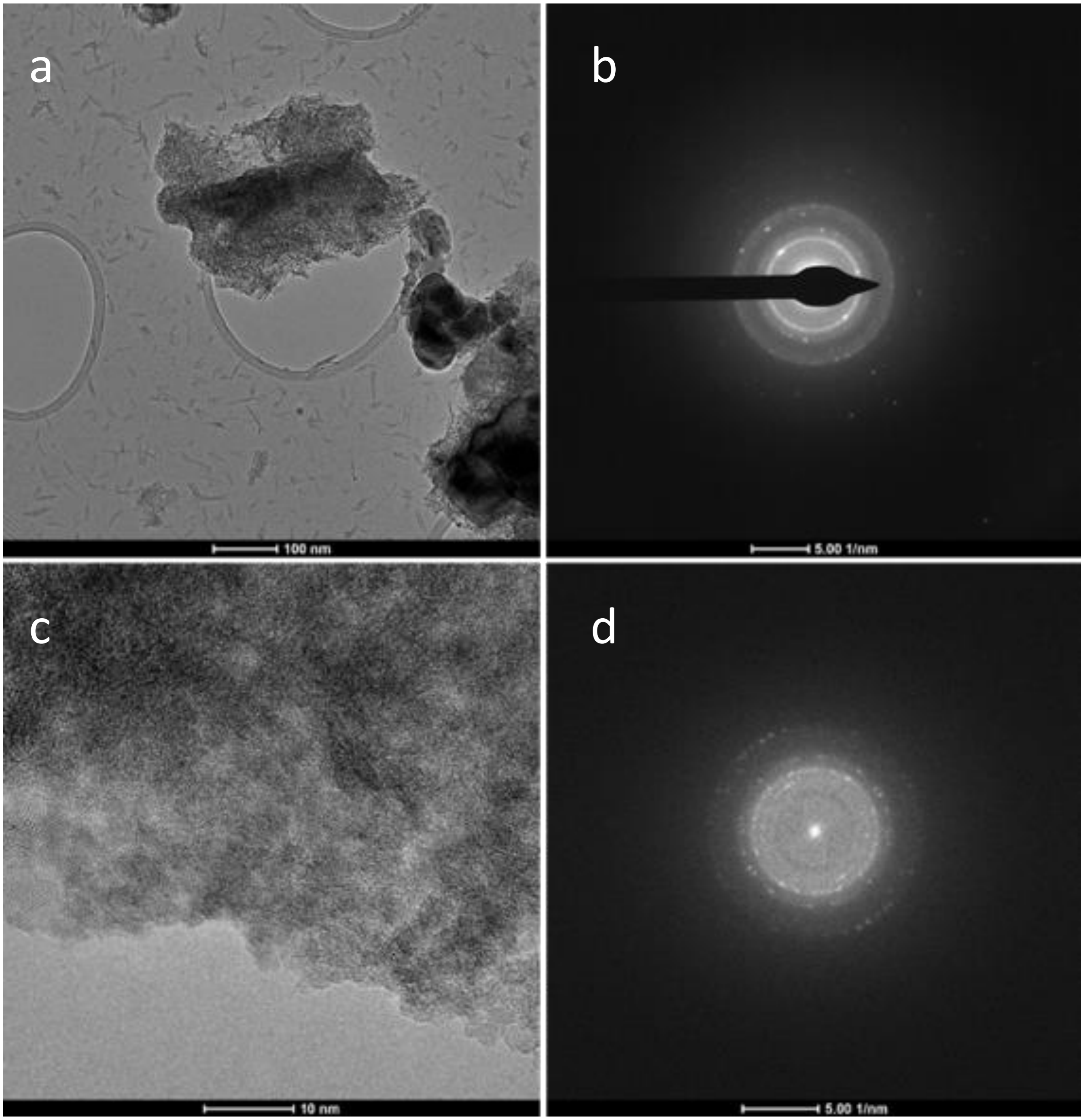

Figure S5. TEM and electron diffraction studies. (a,c) TEM images and (b,d) selected area electron diffraction patterns of nanospindle crystals around the Fe3-xNixO4 particles of FeNiO-250-4. Nanospindles are prevalent on the lacey carbon film of the TEM grid.

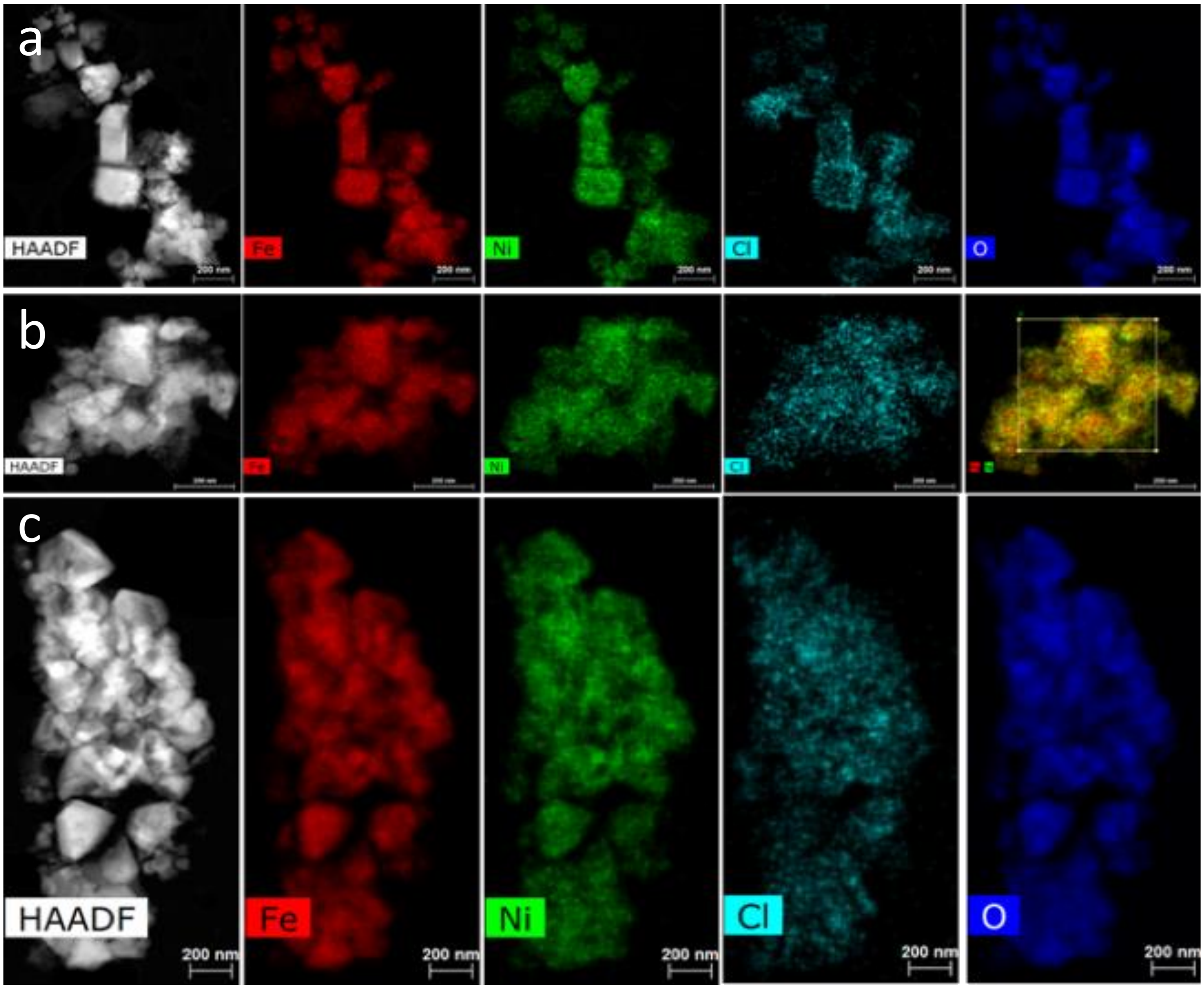

Figure S6. EDS-based elemental mapping analysis. (a-c) EDS mapping studies of FeNiO-250-4. One can see that Cl is rather evenly distributed throughout the samples. Fe and Ni do not show any phase segregation, suggesting a homogeneous distribution within FeNiO-250-4. In the images, the large and uniform crystals are nanoparticles, while the small and uniform structure, with a rich chlorine distribution, are nanospindles.

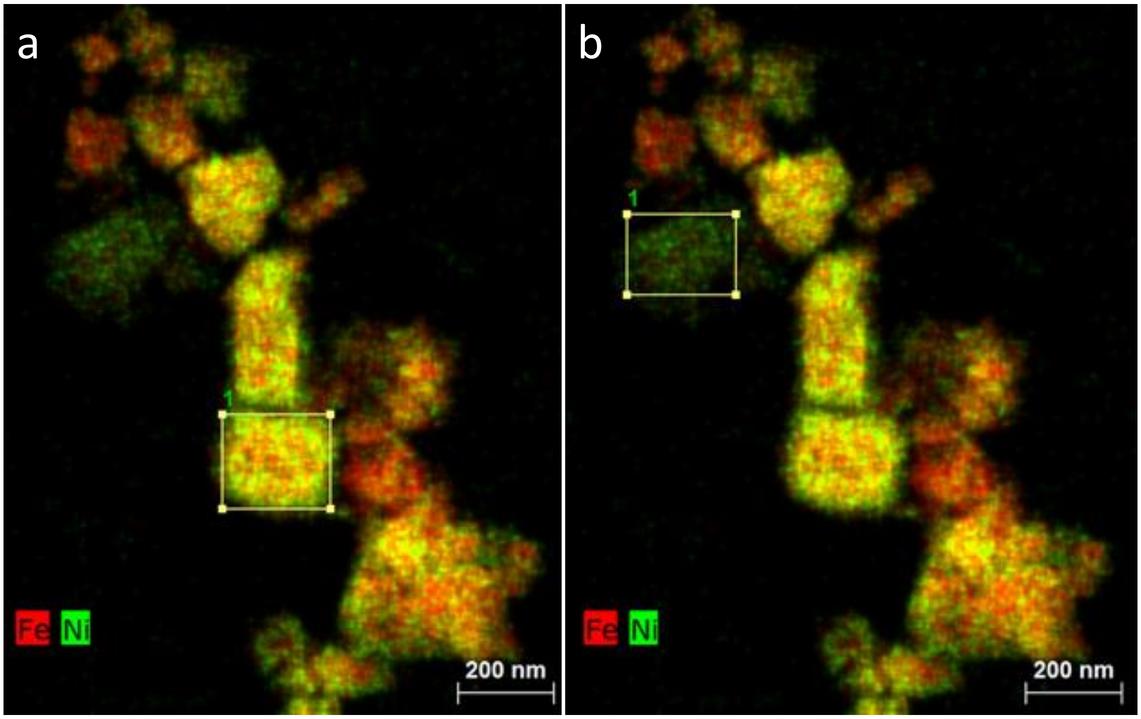

Figure S7. EDS-based elemental mapping analysis. Elemental maps of (a) FeNi oxide spinel nanoparticles and (b) nanospindle area.

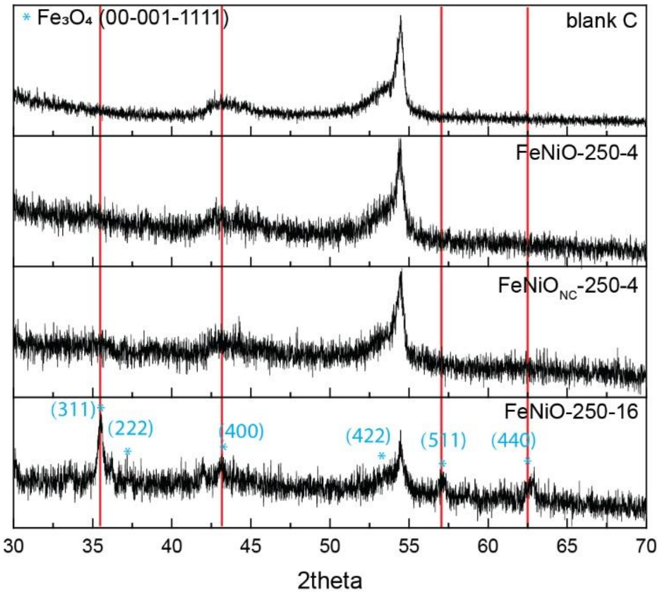

Figure S8. XRD patterns of the sample series. The peak around 2q = 55° is from the carbon background. Notably, no apparent XRD patterns of spinel metal oxide could be resolved with FeNiO-250-4 and $FeNiO_{NC}$-250-4, likely due to the low contents in the samples. By contrast, the FeNiO-250-16 sample exhibited a series of diffraction patterns that matched those of Fe_3O_4 (card 00-001-1111), consistent with the formation of a clear $Fe_{3-x}Ni_xO_4$ spinel structure.

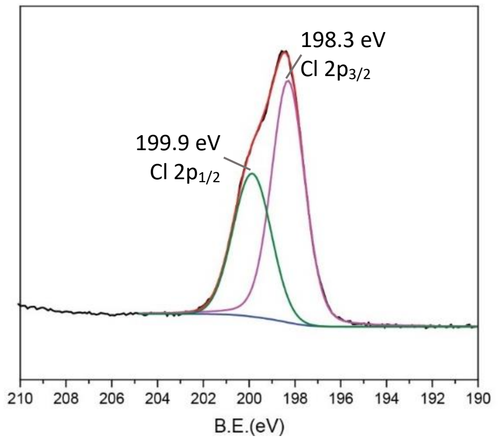

Figure S9. XPS spectrum of the Cl 2p electrons of FeNiO-250-4. The peaks at 198.3 and 199.9 eV are the $2p_{3/2}$ and $2p_{1/2}$ electrons of metal-Cl, respectively. Black curve is experimental data and colored curves are deconvolution fits.

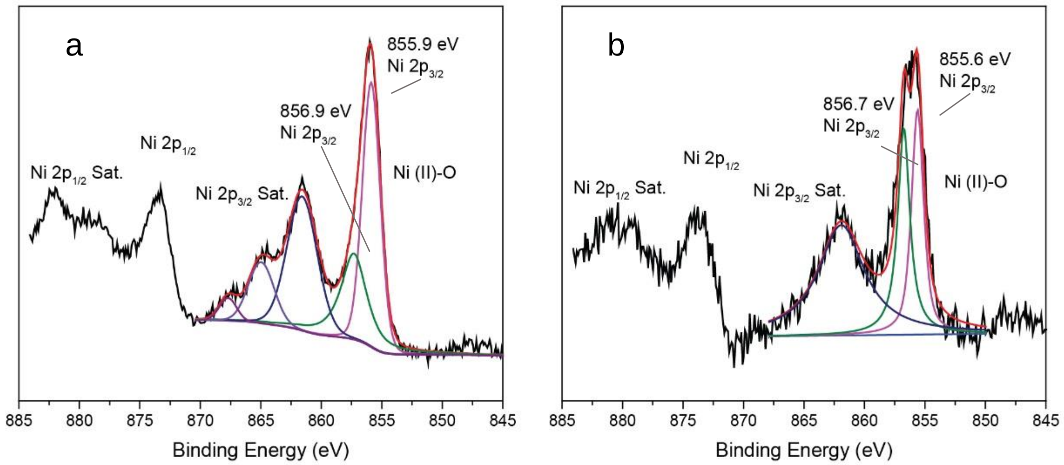

Figure S10. High-resolution XPS spectra of the Ni 2p electrons of (a) $FeNiO_{NC}$-250-4 and (b) FeNiO-250-16. Black curves are experimental data and colored curves are deconvolution fits.

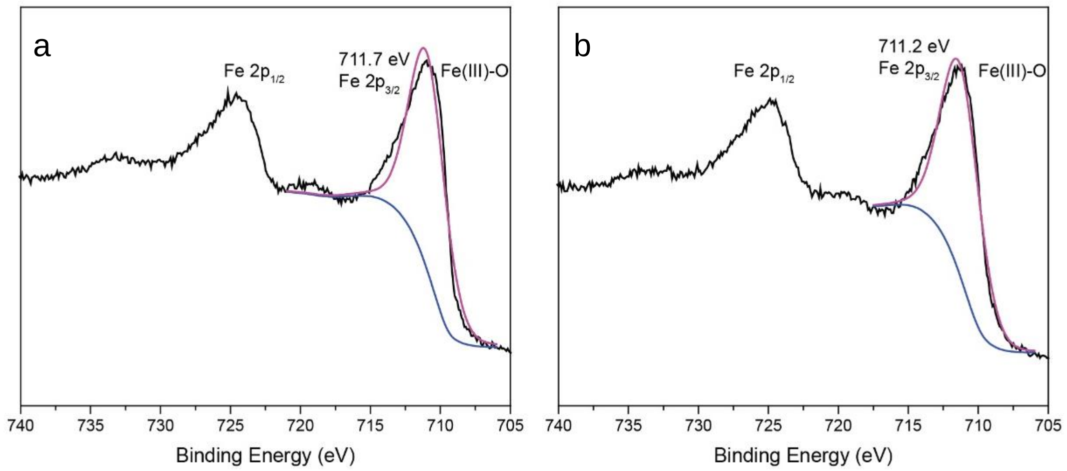

Figure S11. High-resolution XPS spectra of the Fe 2p electrons of (a) $FeNiO_{NC}$-250-4 and (b) FeNiO-250-16. Black curves are experimental data and colored curves are deconvolution fits.

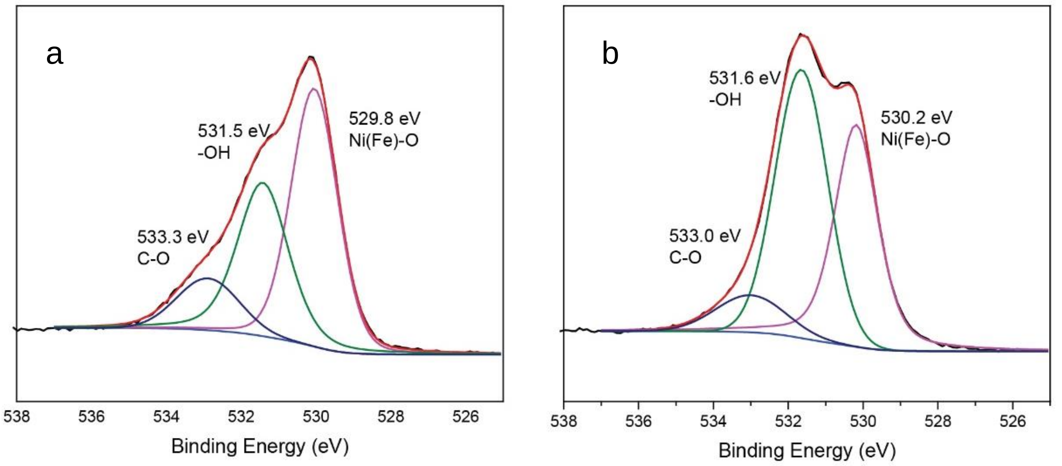

Figure S12. High-resolution XPS scans of the O 1s electrons of a) $FeNiO_{NC}$-250-4 and b) FeNiO-250-16. Black curves are experimental data and colored curves are deconvolution fits.

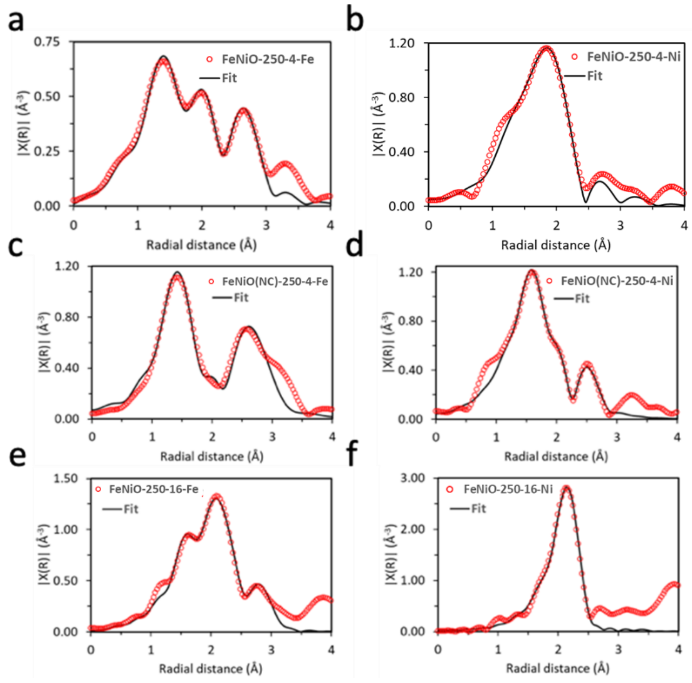

Figure S13. EXAFS fitting results of Fe (left) and Ni (right) for (a, b) FeNiO-250-4, (c, d) $FeNiO_{NC}$-250-4, and (e, f) FeNiO-250-16.

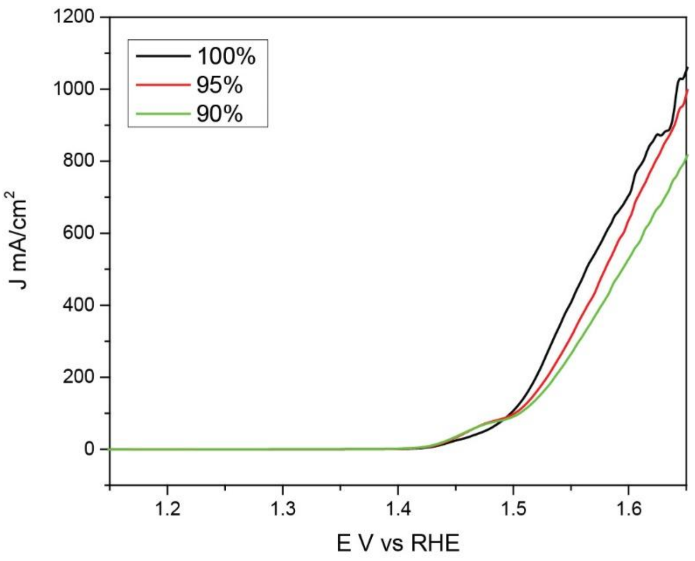

Figure S14. LSV curves of FeNiO-250-4 in 1 M KOH at different levels of iR compensation.

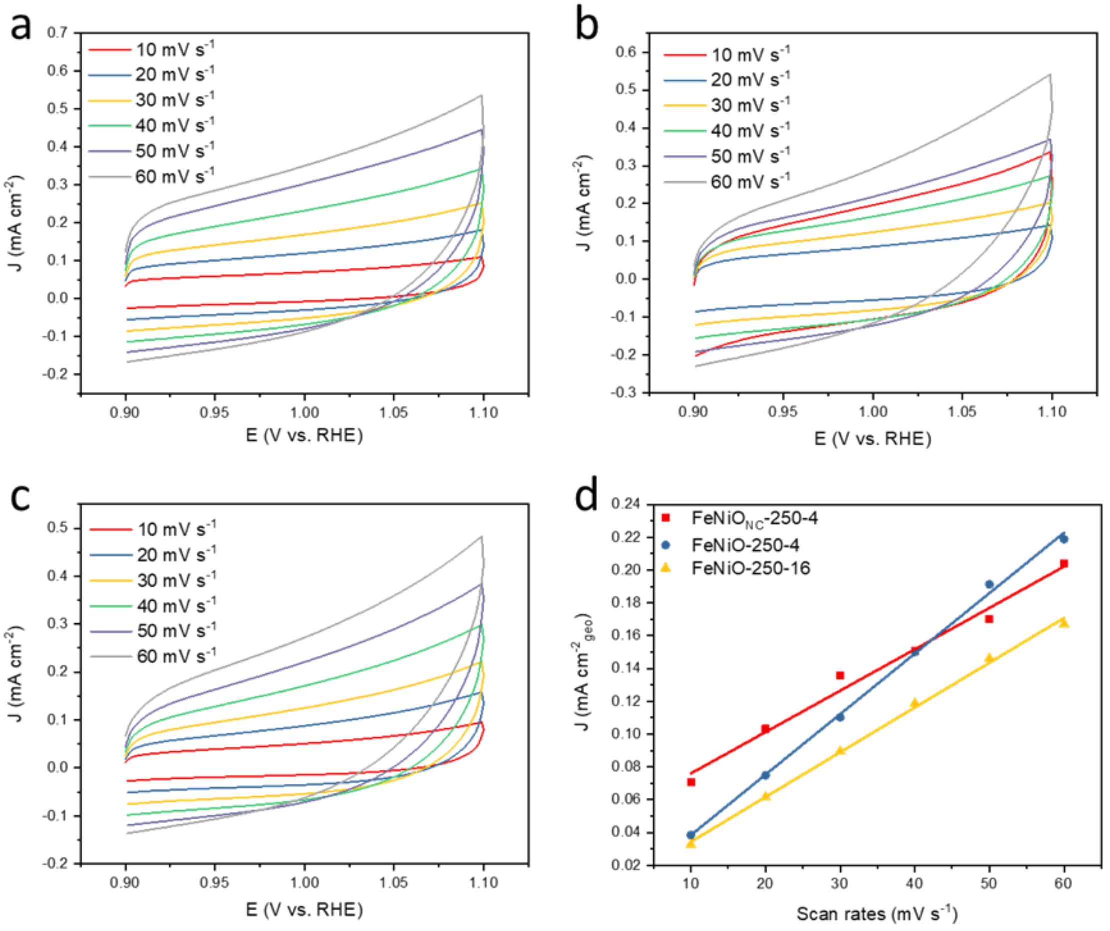

Figure S15. CV curves at difference scan rates for (a) FeNiO-250-4, (b) $FeNiO_{NC}$-250-4, and (c) FeNiO-250-16 in 1 M KOH. (d) The variation of the double-layer charging current with potential scan rate, from which the double-layer capacitance (C_{dl}) is estimated and the corresponding ECSA is calculated by normalizing the C_{dl} to 0.04 mF cm^{-2}, according to reference [*6*].

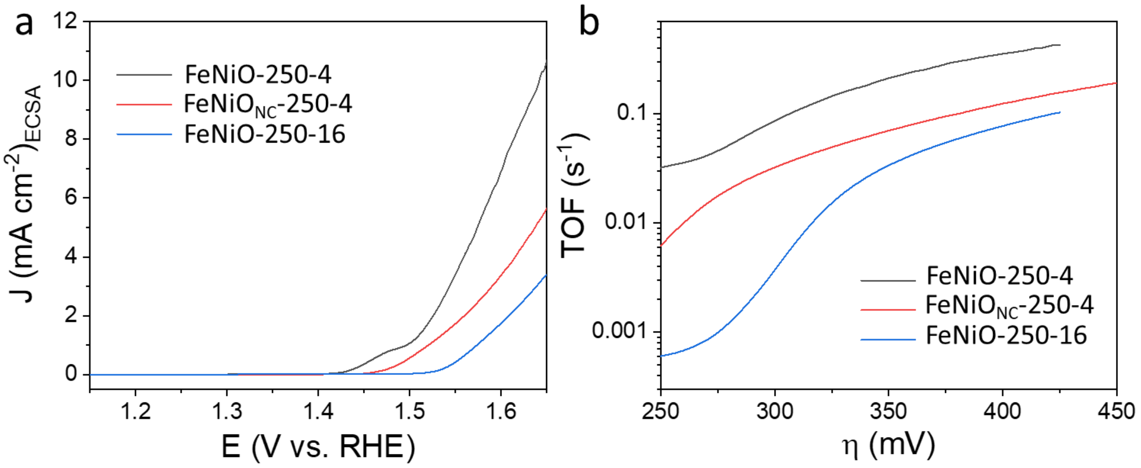

Figure S16. (a) LSV curves normalized to ECSA. (b) TOF curves of FeNiO-250-4, $FeNiO_{NC}$-250-4, and FeNiO-250-16.

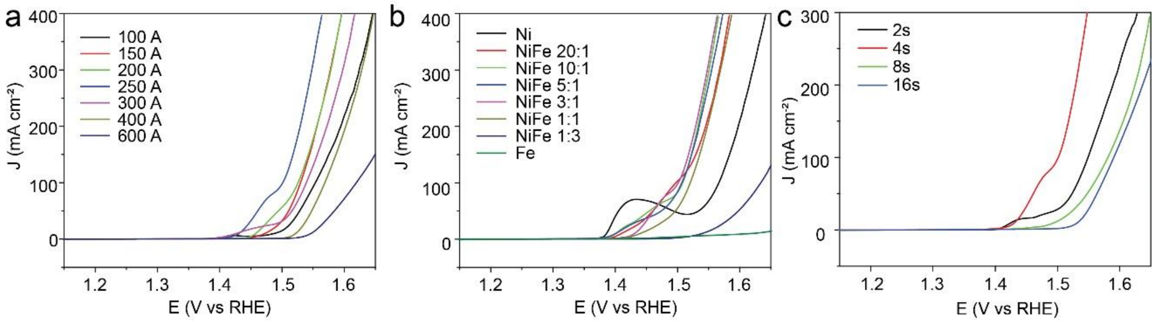

Figure S17. Optimization of OER performance of FeNiO samples. OER polarization curves of (a) FeNiO-X-4 at X = 100, 150, 200, 250, 300, 400, and 600 A, (b) FeNiO-250-4 but at different initial feed ratios of Ni:Fe (1:0, 20:1, 10:1, 5:1, 3:1, 1:1, 1:3, and 0:1), and (c) FeNiO-250-Y at Y = 2, 4, 8, and 16 s.

The induction current of 250 A also represents the optimal condition, as the FeNiO-250-4 clearly outperformed others prepared at different currents, with the OER performance decreasing in order of FeNiO-250-4 > FeNiO-200-4 ~ FeNiO-150-4 > FeNiO-300-4 > FeNiO-100-4 > FeNiO-400-4 > FeNiO-600-4 (panel a). This suggests that the resulting heating temperature of 300°C was most favorable for the conversion of the Ni and Fe precursors to metal oxides and minimization of Fe-Ni phase segregation. The impact of the initial feed ratio on the FeNiO OER performance was also examined, and a Ni:Fe feed ratio of 3:1 was found to be the best (panel b). In panel (c), the activity decreases in the order of FeNiO-250-4 > FeNiO-250-2 > FeNiO-250-8 > FeNiO-250-16. This is because too short a heating time (e.g., 2 s) did not produce a high enough temperature for the conversion of $FeCl_3$ and $NiCl_2$ into FeNiO, while too long a heating time (e.g., 8, and 16 s) could induce severe phase segregation of the metal oxides and depleted Cl in the final product; and 4 s represented the optimal heating time.

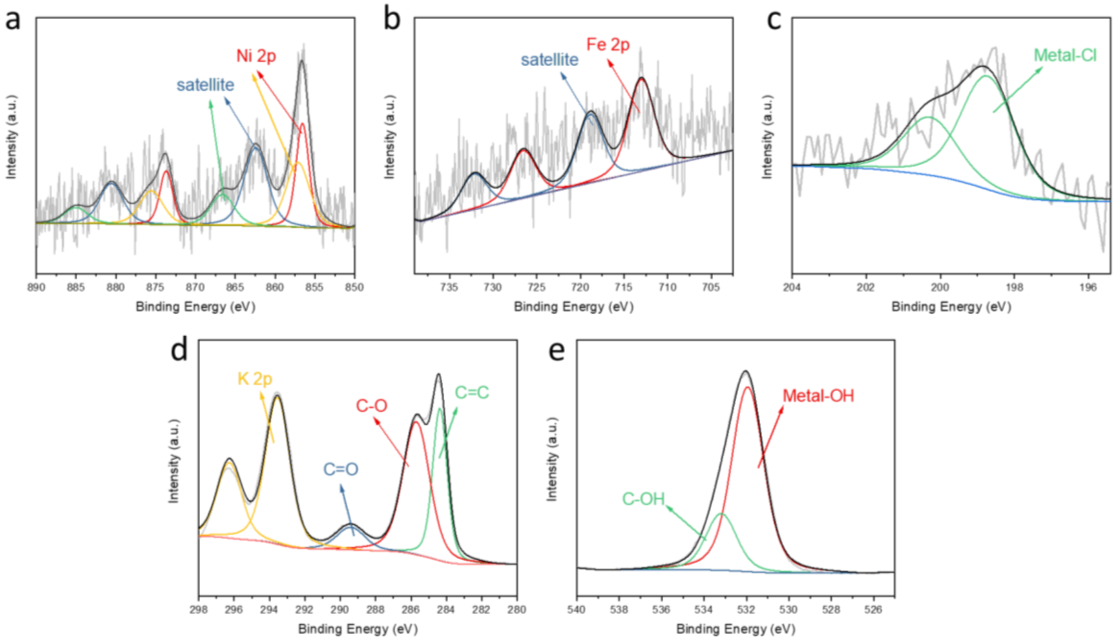

Figure S18. High-resolution XPS scans of the (a) Ni 2p, (b) Fe 2p, (c) Cl 2p, (d) C 1s and (e) O 1s electrosn of FeNiO-250-4 after stability test. Black curves are experimental data and colored curves are deconvolution fits.

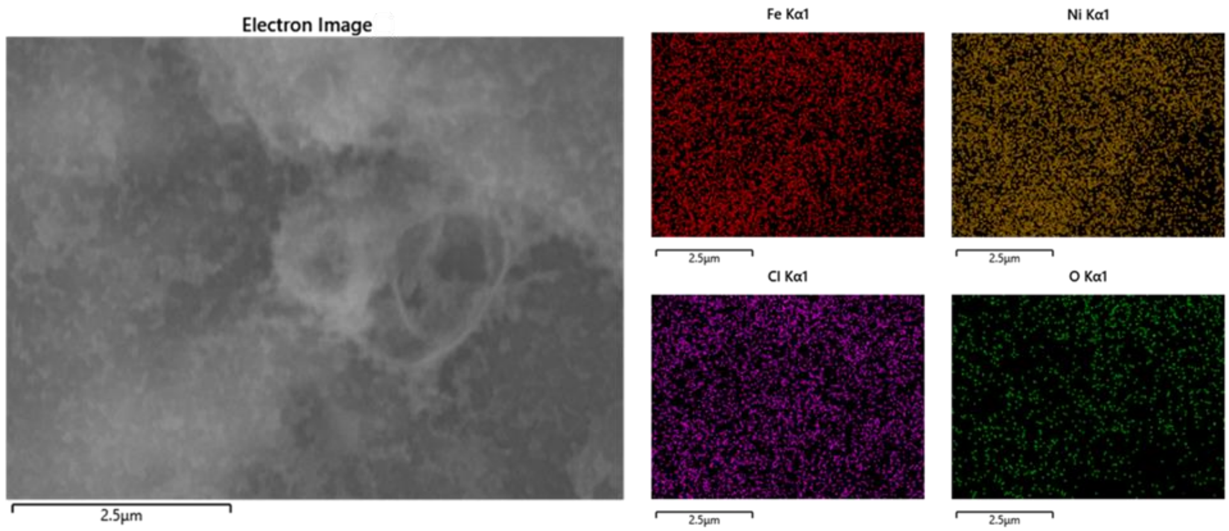

Figure S19. SEM image and the corresponding EDS-based elemental mapping analysis of FeNiO-250-4 after stability test.

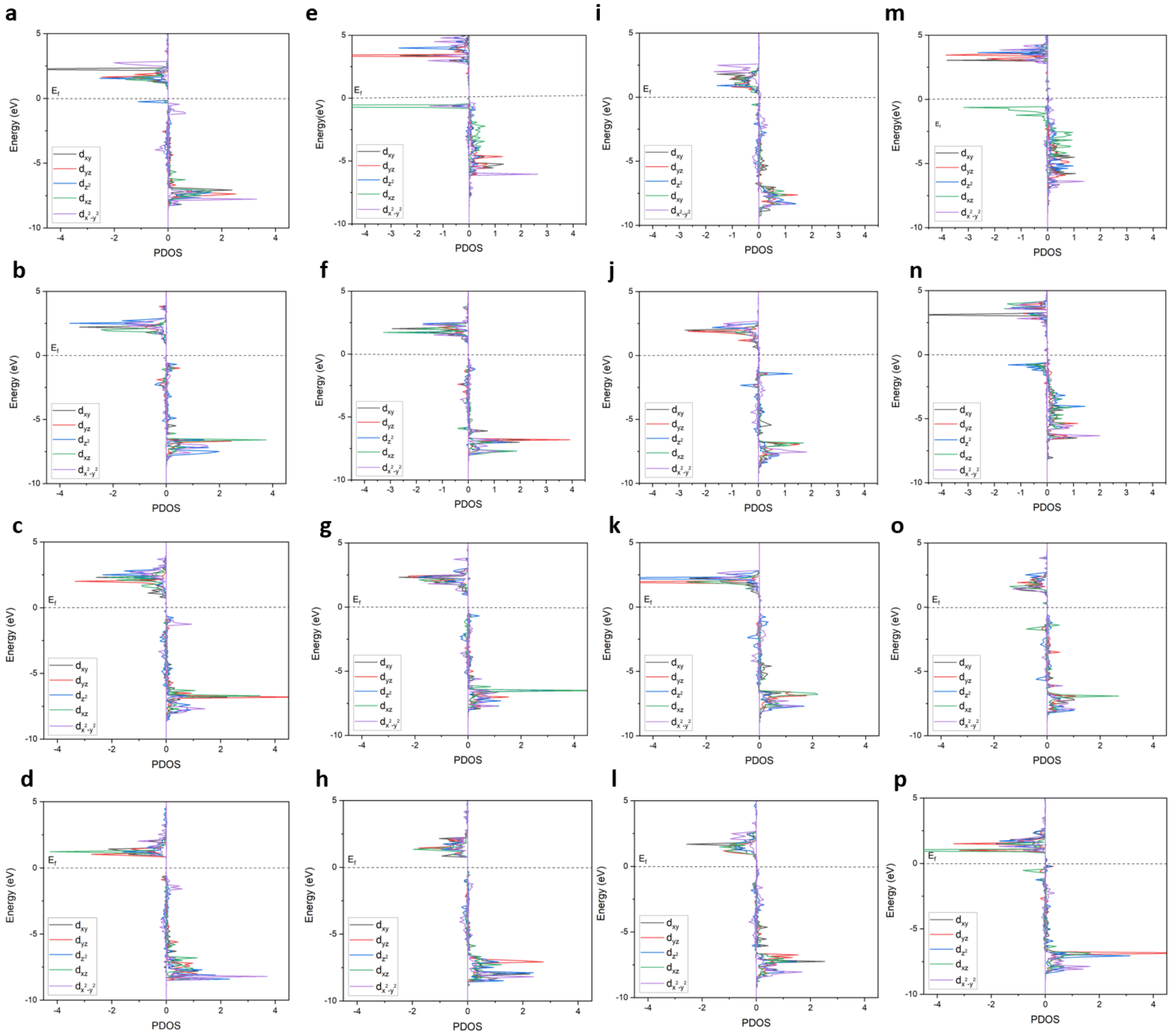

Figure S20. PDOS of the Fe1 atom. (a-d) $Fe(OH)Fe_2O_4$, (e-h) $Fe(OH)Fe_2O_4(Cl)$, (i-l) $Ni(OH)Fe_2O_4$ and (m-p) $Ni(OH)Fe_2O_4$ (Cl). The first row (a, e, I and m) represents PDOS of *–#, the second row (b, f, j, and n) represents PDOS of *–#OH, the third row (c, g, k, and o) represents PDOS of HO*–# OH, and the fourth row (d, h, l and p) represents PDOS of HO*–# O.

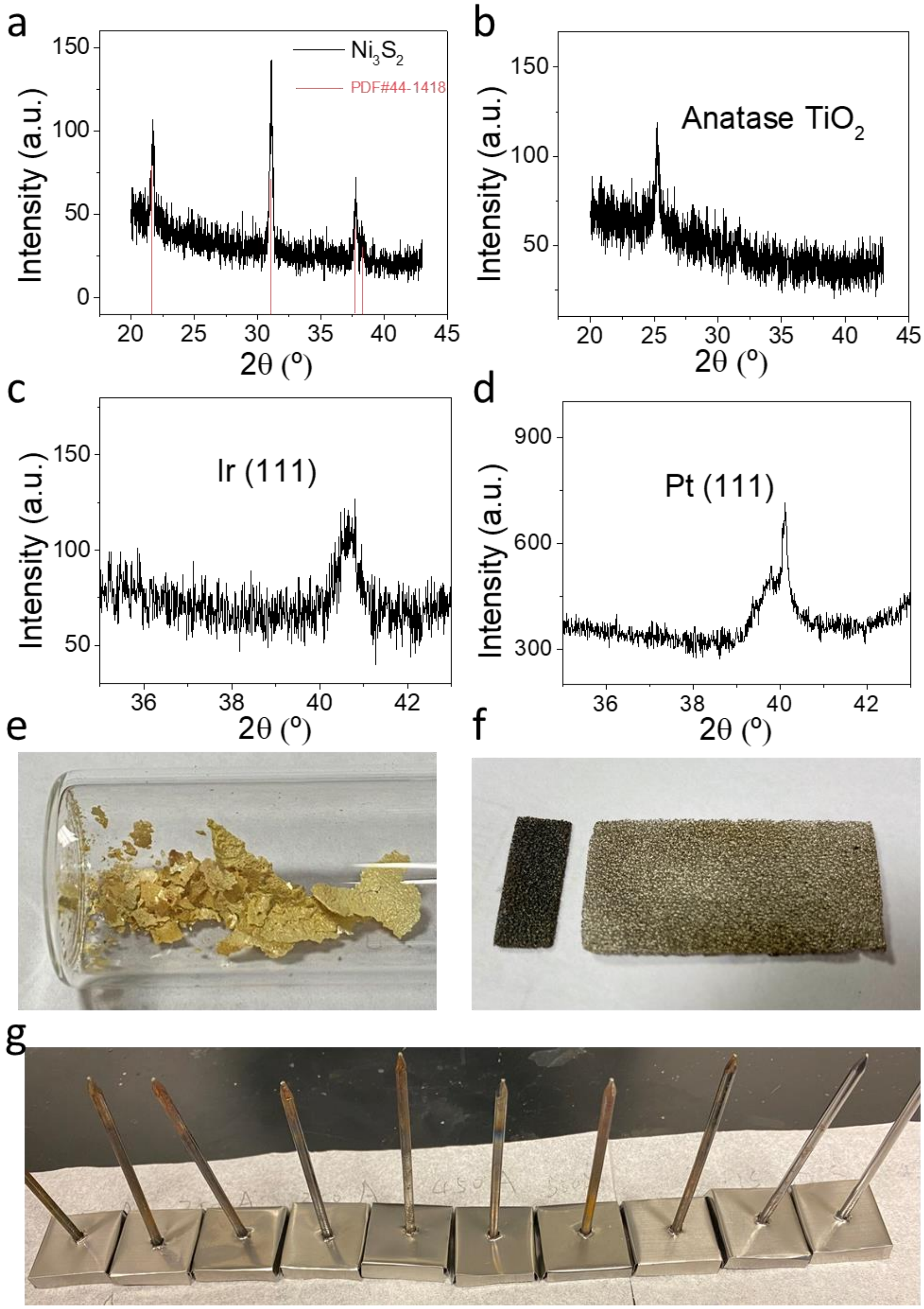

Figure S21. Additional materials prepared by MIHRQ. XRD patterns of (a) Ni_3S_2, (b) TiO_2, (c) Ir, and (d) Pt produced on nickel foam. Photographs of (e) carbon nitride (C_3N_4), (f) nickel foam before (right piece) and after (left piece) induction heating in air, and (g) 10 heating setups for larger-scale synthesis or for the preparationof a diverse range of materials.

The MIHRQ technique can be readily extended to the preparation of a wide range of materials for varied applications (Figure S21), thus offering a new paradigm for materials structural manipulation and

engineering. The nickel foam before and after MIH treatment in air is shown in Figure S17f, where the apparent change of the color appearance signifies ready oxidation of the nickel foam. When a calculated amount of a sulfur-toluene solution or tetrabutyl titanate is dropcast onto the nickel foam, MIH treatment leads to facile production of Ni_3S_2 (Figure S21a) or anatase TiO2 (Figure S21b).

MIHRQ can also be used to prepare noble metal-based samples. For instance, by dropcasting an ethanolic solution of $Ir(acac)_3$ or $Pt(acac)_2$ onto a piece of carbon paper, we successfully prepared carbon-supported Ir (Figure S21c) or Pt nanoparticles (Figure S21d). This method can also treat free-standing powder materials. For example, with melamine loaded into the heating apparatus, graphitic carbon nitride (C_3N_4) can be produced easily (Figure S21e).

Note that the MIHRQ method exhibits a promising outlook for scale-up synthesize. This establishes a solid foundation of industrialization and high-throughput electrode/material synthesis. After attempting the heating elements with different shape, size, thickness, and material, we have successfully normalized the configuration and preparation procedure of the heating element. For instance, with 10 induction heating setups (Figure S21g), we are able to prepare up to 40 samples with different configurations, within just several minutes. We anticipate this method will make significant contributions to material synthesis, structural engineering and applications. Such studies are ongoing and results will be reported in due course.

Table S1. Elemental contents of the sample series based on EDS measurements

element	at%			
	FeNiO-250-4	FeNiO-250-4 (spindle)	$FeNiO_{NC}$-250-4	FeNiO-250-16
Fe	37.01	20.99	4.04	40.06
O	51.77	54.89	61.18	52.39
Ni	8.95	11.13	26.22	6.11
Cl	1.96	12.01	0.95	0.17

Table S2. Fitting results of the EXAFS data of FeNiO-250-4

bond	CN	R (Å)	σ^2 (Å^2) × 10^{-3}	E_0 (eV)	R factor
Fe-O	3.3(7)	1.98(3)	8(4)	0(2)	0.0266
Fe-Cl	2.1(6)	2.43(3)	8(4)	0(2)	0.0266
Fe-Fe	1.9(7)	3.01(3)	8(4)	0(2)	0.0266
Ni-O	2.7(7)	2.03(5)	3(6)	-3(4)	0.0266
Ni-Cl	3(1)	2.40(4)	3(6)	-3(4)	0.0266

Table S3. Fitting results of the EXAFS data of $FeNiO_{NC}$-250-4

bond	CN	R (Å)	σ^2 (Å^2) × 10^{-3}	E_0 (eV)	R factor
Fe-O	4.2(9)	1.97(3)	7(4)	0(2)	0.0294
Fe-Fe	4(1)	3.04(3)	7(4)	0(2)	0.0294
Fe-Cl	0.7(5)	2.44(4)	7(4)	0(2)	0.0294
Ni-O	4.7(5)	2.05(1)	6(2)	-3(1)	0.0060
Ni-Cl	1.5(3)	2.39(2)	6(2)	-3(1)	0.0060
Ni-Ni	1.6(8)	2.95(2)	9(4)	-3(1)	0.0060

Table S4. Fitting results of the EXAFS data of FeNiO-250-16

bond	CN	R (Å)	σ^2 (Å^2) × 10^{-3}	E_0 (eV)	R factor
Fe-O	2.0(5)	1.98(2)	3(3)	3(2)	0.0113
Fe-Cl	1.2(4)	2.30(3)	3(3)	3(2)	0.0113
Fe-Fe	3(1)	2.57(2)	7(3)	3(2)	0.0113
Fe-Fe	1.9(6)	3.13(3)	7(3)	3(2)	0.0113
Ni-O	2(1)	2.02(3)	6(9)	-7(2)	0.0249
Ni-Ni	8(1)	2.50(1)	6(1)	-7(2)	0.0249

Table S5. Comparison with relevant Fe-Ni oxide-based OER catalysts reported in the literature

Initial material	Surface	Catalytic activity	Tafel slope (mV dec^{-1})	Current density (mA cm^{-2})	η (mV)
Quantum dots $NiFe_2O_4$[*7*]		Oxygen vacancy	37	10	262
$NiFe_2O_4$[*2*]	(Ni,Fe)OOH	(Ni)*OH 2.05 eV	40	100	309
$NiFe_2O_4$[*8*]	Defect's formation	Oxygen vacancy	40	10	350
V-dopped $NiFe_2O_4$[*9*]			43.9	10	271.3
$NiFe_2O_4$ under layer Ni_3Fe[*1*]		*O → *OOH RDS	52.3 (TON =0.27/s) ~ 5* NiFe2O4		
$NiFe_2O_4$ NP/NiFe LDH[*10*]			28	10	300
ɣ-FeOOH on ɣ-NiOOH (support)[*11*]			34	100 (10)	248 (215)
PA-Gd-$Ni(OH)_2Cl$ (partially alkylated gadolinium-doped nickel oxychloride)[*12*]			40	10	220
Ruddlesden–Popper-type oxychloride[*13*]				10	300
FeNi LDH thin film on Fe foam[*14*]	Layered Double hydroxide		48.3	1000 (500)	340 (300)
$Cu_{1-x}NNi_{3-y}$@FeNiCu (oxy)hydroxide[*15*]	FeNiCu (oxy)hydroxide		45	10	280
FeCoCrNi alloy film[*16*]	Metal oxyhydroxides	Ni^{4+} sites	38.7	10	304
Ni-Fe hydroxide[*17*]	Fe-Ni hydroxides	Fe/NiOOH		100	~340
$NiFe_x$ molecules on Hetero-atom doped graphene[*18*]	Ni-Fe with OH^- ions	Ni-Fe hydroxides	39	10	310
Ni/NiO-NF[*19*]	$Ni^{III/IV}$	photogenerated holes	41	100 (20)	380 (260)
MoFe:$Ni(OH)_2$[*20*]	Mo, Fe, NiOOH		47	100	280
Fe doped NiO_x nanotubes[*21*]	NiO_x	Ni vacancy	49	10	310

Table S6. Electrochemical surface areas of the sample series

Sample	C_{dl} (mF cm^{-2})$_{geo}$	C_{dl} (mF)	ECSA (cm^2)
FeNiO$_{NC}$-250-4	2.52	0.63	15.75
FeNiO-250-4	3.69	0.92	23.06
FeNiO-250-16	2.73	0.68	17.06

Movie S1. Magnetic induction heating at a solenoid current of 200 A for a heating time of 4 s.

Movie S2. Magnetic induction heating at a solenoid current of 600 A for a heating time of 4 s.

Movie S3. Oxygen evolution catalyzed by FeNi-250-4.

References

[1] M. Alharthy, M. H. Suliman, A.-R. Al-Betar *et al.*, Reaping the catalytic benefits of both surface (NiFe2O4) and underneath (Ni3Fe) layers for the oxygen evolution reaction. *Sustainable Energy & Fuels,* vol. 5, no. 10, pp. 2704-2714, 2021.

[2] L. Gao, X. Cui, Z. Wang *et al.*, Operando unraveling photothermal-promoted dynamic active-sites generation in NiFe2O4 for markedly enhanced oxygen evolution. *Proceedings of the National Academy of Sciences,* vol. 118, no. 7, pp. 2021.

[3] I. C. Man, H. Y. Su, F. Calle-Vallejo *et al.*, Universality in oxygen evolution electrocatalysis on oxide surfaces. *ChemCatChem,* vol. 3, no. 7, pp. 1159-1165, 2011.

[4] M. Tang, Q. Ge, Mechanistic understanding on oxygen evolution reaction on γ-FeOOH (010) under alkaline condition based on DFT computational study. *Chinese Journal of Catalysis,* vol. 38, no. 9, pp. 1621-1628, 2017.

[5] J. K. Nørskov, J. Rossmeisl, A. Logadottir *et al.*, Origin of the overpotential for oxygen reduction at a fuel-cell cathode. *The Journal of Physical Chemistry B,* vol. 108, no. 46, pp. 17886-17892, 2004.

[6] C. C. L. McCrory, S. Jung, J. C. Peters *et al.*, Benchmarking Heterogeneous Electrocatalysts for the Oxygen Evolution Reaction. *Journal of the American Chemical Society,* vol. 135, no. 45, pp. 16977-16987, 2013.

[7] H. Yang, Y. Liu, S. Luo *et al.*, Lateral-Size-Mediated Efficient Oxygen Evolution Reaction: Insights into the Atomically Thin Quantum Dot Structure of NiFe2O4. *ACS Catalysis,* vol. 7, no. 8, pp. 5557-5567, 2017.

[8] Q. Yue, C. Liu, Y. Wan *et al.*, Defect engineering of mesoporous nickel ferrite and its application for highly enhanced water oxidation catalysis. *Journal of Catalysis,* vol. 358, pp. 1-7, 2018.

[9] R. Wei, X. Bu, W. Gao *et al.*, Engineering surface structure of spinel oxides via high-valent vanadium doping for remarkably enhanced electrocatalytic oxygen evolution reaction. *ACS applied materials & interfaces,* vol. 11, no. 36, pp. 33012-33021, 2019.

[10] Z. Wu, Z. Zou, J. Huang *et al.*, NiFe2O4 Nanoparticles/NiFe Layered Double-Hydroxide Nanosheet Heterostructure Array for Efficient Overall Water Splitting at Large Current Densities. *ACS Applied Materials & Interfaces,* vol. 10, no. 31, pp. 26283-26292, 2018.

[11] F. Song, M. M. Busch, B. Lassalle-Kaiser *et al.*, An Unconventional Iron Nickel Catalyst for the Oxygen Evolution Reaction. *ACS Central Science,* vol. 5, no. 3, pp. 558-568, 2019.

[12] T. u. Haq, Y. Haik, I. Hussain *et al.*, Gd-Doped Ni-Oxychloride Nanoclusters: New Nanoscale Electrocatalysts for High-Performance Water Oxidation through Surface and Structural Modification. *ACS Applied Materials & Interfaces,* vol. 13, no. 1, pp. 468-479, 2021.

[13] Y. Miyahara, T. Fukutsuka, T. Abe *et al.*, Dual-Site Catalysis of Fe-Incorporated Oxychlorides as Oxygen Evolution Electrocatalysts. *Chemistry of Materials,* vol. 32, no. 19, pp. 8195-8202, 2020.

[14] Y. P. Liu, X. Liang, L. Gu *et al.*, Corrosion engineering towards efficient oxygen evolution electrodes with stable catalytic activity for over 6000 hours. *Nature Communications,* vol. 9, pp. 2018.

[15] Y. P. Zhu, G. Chen, Y. J. Zhong *et al.*, A surface-modified antiperovskite as an electrocatalyst for water oxidation. *Nature Communications,* vol. 9, pp. 2018.

[16] N. Zhang, X. B. Feng, D. W. Rao *et al.*, Lattice oxygen activation enabled by high-valence metal sites for enhanced water oxidation. *Nature Communications,* vol. 11, no. 1, pp. 2020.

[17] C. G. Kuai, Z. R. Xu, C. Xi *et al.*, Phase segregation reversibility in mixed-metal hydroxide water oxidation catalysts. *Nature Catalysis,* vol. 3, no. 9, pp. 743-753, 2020.

[18] J. Wang, L. Y. Gan, W. Y. Zhang *et al.*, In situ formation of molecular Ni-Fe active sites on heteroatom-doped graphene as a heterogeneous electrocatalyst toward oxygen evolution. *Science Advances,* vol. 4, no. 3, pp. 2018.

[19] X. L. Han, Y. F. Yu, Y. Huang *et al.*, Photogenerated Carriers Boost Water Splitting Activity over Transition-Metal/Semiconducting Metal Oxide Bifunctional Electrocatalysts. *Acs Catalysis,* vol. 7, no. 10, pp. 6464-6470, 2017.

[20] Y. S. Jin, S. L. Huang, X. Yue *et al.*, Mo- and Fe-Modified Ni(OH)(2)/NiOOH Nanosheets as Highly Active and Stable Electrocatalysts for Oxygen Evolution Reaction. *Acs Catalysis,* vol. 8, no. 3, pp. 2359-2363, 2018.

[21] G. Wu, W. X. Chen, X. S. Zheng *et al.*, Hierarchical Fe-doped NiOx nanotubes assembled from ultrathin nanosheets containing trivalent nickel for oxygen evolution reaction. *Nano Energy,* vol. 38, pp. 167-174, 2017.
